# Diverse WGBS profiles of longissimus dorsi muscle in Hainan black goats and hybrid goats

**DOI:** 10.1186/s12863-023-01182-x

**Published:** 2023-12-14

**Authors:** Yuwei Ren, Xing Chen, Xinli Zheng, Feng Wang, Ruiping Sun, Limin Wei, Yan Zhang, Hailong Liu, Yanning Lin, Lingling Hong, Xiaoxian Huang, Zhe Chao

**Affiliations:** 1 Key Laboratory of Tropical Animal Breeding and Disease Research, Institute of Animal Science and Veterinary Medicine, Hainan Academy of Agricultural Sciences, Haikou, 571100 China; 2 Institute of Animal Husbandry and Veterinary, Wuhan Academy of Agricultural Science, Wuhan, 430000 China

**Keywords:** Goats, Whole-genome bisulfite sequencing, DNA methylation, Muscle fiber, Vasodilatation, Glycogen storage

## Abstract

**Background:**

Goat products have played a crucial role in meeting the dietary demands of people since the Neolithic era, giving rise to a multitude of goat breeds globally with varying characteristics and meat qualities. The primary objective of this study is to pinpoint the pivotal genes and their functions responsible for regulating muscle fiber growth in the longissimus dorsi muscle (LDM) through DNA methylation modifications in Hainan black goats and hybrid goats.

**Methods:**

Whole-genome bisulfite sequencing (WGBS) was employed to scrutinize the impact of methylation on LDM growth. This was accomplished by comparing methylation differences, gene expression, and their associations with growth-related traits.

**Results:**

In this study, we identified a total of 3,269 genes from differentially methylated regions (DMR), and detected 189 differentially expressed genes (DEGs) through RNA-seq analysis. Hypo DMR genes were primarily enriched in KEGG terms associated with muscle development, such as MAPK and PI3K-Akt signaling pathways. We selected 11 hub genes from the network that intersected the gene sets within DMR and DEGs, and nine genes exhibited significant correlation with one or more of the three LDM growth traits, namely area, height, and weight of loin eye muscle. Particularly, *PRKG1* demonstrated a negative correlation with all three traits. The top five most crucial genes played vital roles in muscle fiber growth: *FOXO3* safeguarded the myofiber’s immune environment, *FOXO6* was involved in myotube development and differentiation, and *PRKG1* facilitated vasodilatation to release more glucose. This, in turn, accelerated the transfer of glucose from blood vessels to myofibers, regulated by *ADCY5* and *AKT2*, ultimately ensuring glycogen storage and energy provision in muscle fibers.

**Conclusion:**

This study delved into the diverse methylation modifications affecting critical genes, which collectively contribute to the maintenance of glycogen storage around myofibers, ultimately supporting muscle fiber growth.

**Supplementary Information:**

The online version contains supplementary material available at 10.1186/s12863-023-01182-x.

## Introduction

The domestication of goats (*Capra hircus*) has a long history dating back to the Neolithic era, providing humans with essential resources such as meat, skin, and milk [1]. Goat meat, known for its nutrient-rich composition characterized by low lipids, low cholesterol, and high-quality proteins, has garnered increasing attention from consumers due to its quality. Over time, goat populations have expanded significantly during the process of domestication, resulting in the rapid emergence of numerous goat breeds worldwide. Some of these breeds have demonstrated rapid growth, while others are renowned for their flavors and nutritional value [2]. Throughout the breeding process, it has become evident that cross bred goats inherit advantageous traits from their parent breeds. One key factor influencing muscle quality is glycogen, a crucial component for myocyte growth and nutritional maintenance. Notably, excessive glycogenolysis during slaughter leads to the production of pale, soft, and exudative meat [3]. It has also been recognized that, methylation can impact muscle development [4], although the specific effect of methylation on myocyte growth mediated by glycogen storage in cross bred goats remains unclear.

Muscle glycogen serves not only as an energy source, but also as a regulator of whole-body energy metabolism [5], For instance, bodybuilders manipulate their carbohydrate intake to increase muscle thickness, thus enhancing cross-sectional area, muscle volume, and overall body weight [6]. Conversely, a decrease in muscle glycogen content, as seen during the post-transportation rest period in alpacas before slaughter, can lead to increased drip loss and purge [7], underscoring the significance of glycogen for maintaining meat quality. Moreover, methylation has been linked to porcine meat pH [8], and lamb meat quality [4]. Whole-genome bisulfite sequencing (WGBS) has proven to be a valuable tool for exploring methylation in various tissues due to its ability to provide comprehensive information about methylcytosine [9]. Methylation levels can impact numerous physical functions, including growth, productive performance, and meat quality [10], as well as muscle development [11]. Furthermore, hypomethylated regions found in promoters and enhancers have the potential to enhance the overall health of ovine tissues, leading to increased production of meat, milk, and wool [12]. The GTPase regulator activation has been identified as valuable in differentially methylated regions (DMRs), as it is closely associated with oxidative and glycolytic muscles [13]. Importantly, differences in DNA methylation levels between breeds can result in various production capacities, such as milk yield and muscle development [14]. Methylation has also been linked to meat tenderness through its influence on alternative splicing of muscle genes [15]. Conversely, DNA methylation might cause insulin resistance by affecting lipid droplets and metabolism genes in muscle [16].

Nubian goats, widely distributed in arid regions of Northern Sudan [17], are characterized by rapid growth, excellent meat quality, and high meat production [18]. These qualities make them superior to many local goat breeds in other countries, leading to their use in hybridization programs to improve growth traits. For example, the F1 hybrid offspring resulting from the crossing of Yunling black goats and Nubian goats, have exhibited specific genes expression related to bone development, muscle cell differentiation, lipid metabolism, and adaptation to hot and humid environments [19]. In contrast, Hainan black goats, a unique local breed exclusive to the tropical island in China, have evolved under the distinct climatic conditions of high temperature and high humidity. They are known for their tender meat, high nutritional value, and disease resistance [20]. However, their growth rate and body size are smaller than those of Nubian goats. Hainan black goats typically reach sexual maturity between 4 and 6 months of age and only give birth to one kid per year [21]. Nubian goats were chosen as ideal crossbreeding partners for Hainan black goats to address their subpar reproductive performance and preserve high-quality meat traits.

Furthermore, hybridization involving geographically different species has yielded significant growth advantages in recent years, with hybrid goats (Nubian goats × Hainan black goats), displaying significantly greater weight compared to Hainan black goats at the same age. While the concept of heterosis is well-established, the genetic mechanism responsible for the expression of desirable traits remains unclear, as do the effects of DNA methylation on skeletal muscle and gene expression patterns. Compared with growth traits of other livestock species, such as cattle [22], pigs [23], and sheep [24], the investigation of DNA methylation in goat has been relatively limited to reproductive performance [25, 26]. Therefore, it is imperative to explore the regulatory mechanisms related to DNA methylation in goat growth traits, particularly in the context of glycogen storage in hybrid goat muscle. The primary aim of this study is to identify the key genes responsible for regulating the growth of the longissimus dorsi muscle (LDM) and to assess the effects of DNA methylation on muscle glycogen storage in hybrid goats.

## Results

### Identification and distribution of mC levels

**Fig. 1:**
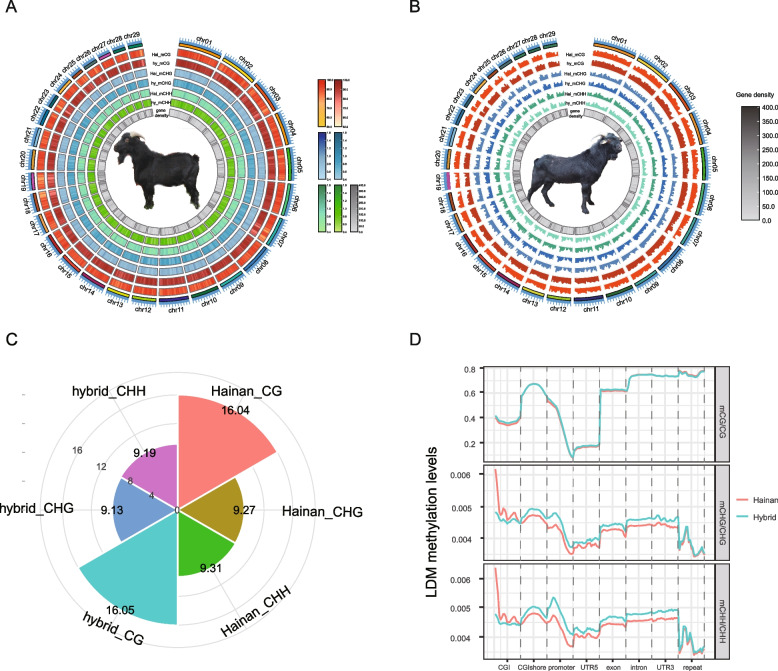
Overview of mC contexts in LDM of Hainan black goats and hybrid goats. **A** Density and (**B**) levels of mC within three contexts (CG, CHG, CHH) across the genome of LDM tissues in two goat species, with Hainan black goats at the center in **A** and hybrid goats in **B**, from innermost to outermost, represent gene density, hybrid mCHH, Hainan mCHH, hybrid mCHG, Hainan mCHG, hybrid mCG, Hainan CG. **C** Comparison of the average log_2_^(mC number)^ in three mC contexts between Hainan black goats and hybrid goats, with the concentric circles indicating log_2_^(mC number)^ values of 0, 4, 8, 12, and 16. **D** Mean mC levels within the three contexts (CG, CHG, and CHH) in various genomic functional regions (promoter, exon, intron, CGI, CGI shore, 3’UTR, 5’UTR, repeat) for LDM tissues in both goat species

WGBS was conducted on LDM tissues from six individuals, and yielded approximate 553.21 Gb of raw data across 1,844,041,561 raw reads, with an average sequencing depth of ~30× for each sample (Table S1). After trimming adaptors and removing low-quality reads, the clean data were mapped to the reference genome (ARS1) to obtain an average mapping ratio of 81.08% (Table S2). The sequencing depth was most often seen to be 24× (Fig. S1), with the highest coverage being 50× (Fig. S2). In general, the methylation density and level of the three mC contexts (CpG, CHG, and CHH) were consistent in LDM between Hainan black goats and hybrid goats (Fig. 1A and B). The CG context occupied a greater relevance on chromosomes 1 compared to other chromosomes (Fig. S3), meanwhile, the CG sites exhibited the largest number and highest methylation proportion across the three mC contexts in Hainan black goats and hybrid goats (Fig. 1C and Fig. S4), as well as methylation levels (Fig. S5).

The average mC levels in genomic functional regions (promoter, exon, intron, CGI, CGI shore, 3’UTR, 5’UTR, repeat) were consistent across the two species. The mCHG and mCHH displayed a little fluctuation in all functional regions of the LDM in Hainan black goats and hybrid goats, while mCG exhibited a gradual decline in the promoter region and relatively lower levels in the 5’UTR region (Fig. 1D and Fig. S6). Additionally, a higher methylation level of mCG was observed within the gene body (exon, intron) and downstream (3’UTR) regions in both goat species (Fig. 1D and Fig. S6).

### The identification and annotation of DMR

**Fig. 2:**
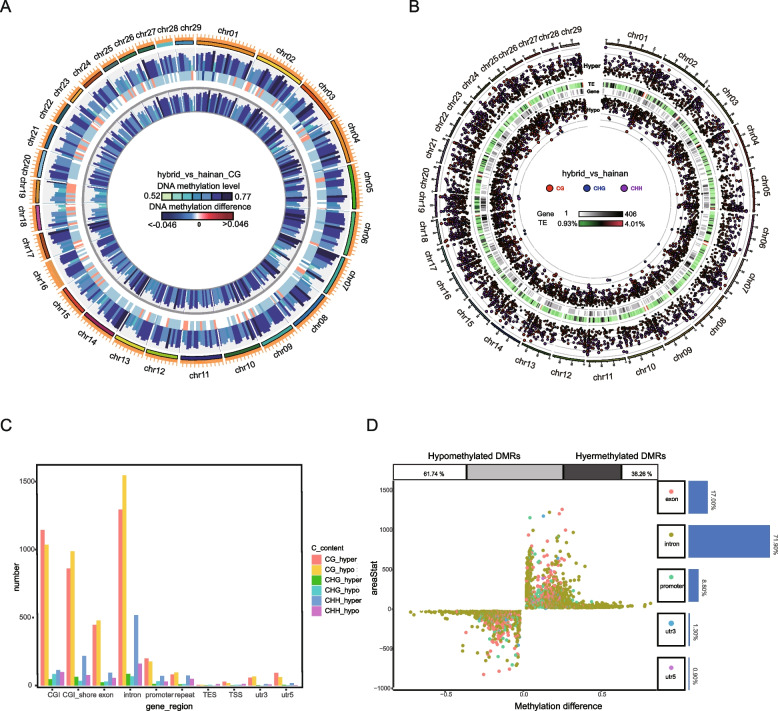
The DMR analysis between Hainan black goats and hybrid goats. **A** The outermost layer and innermost layers of the circle depict DNA methylation levels for the CG content in hybrid goats and Hainan black goats, respectively. The middle layer represents differences in DNA methylation levels between the two goat species. Each bin in the circos figure was 6,553,600bp. Dark blue bars indicate significantly lower (*p* < 0.05) DNA methylation levels in hybrid goats compared to Hainan black goats, while red bars signify significantly higher (*p* < 0.05) DNA methylation levels. **B** The outer and the inner layers distinguish between hyper and hypo DMR, while the two middle layers show the density of TE and genes. The small red, blue, and purple circles represent CG, CHG, and CHH contexts, respectively. The position of these circles relative to the center indicated the magnitude of the difference in methylation levels (diff.methylation) within DMRs. The size of the circles corresponds to the extent of the difference (areaStat) within DMRs. **C** Distribution of genes within DMRs across various genomic functional regions. **D** Comparison of gene functional regions between hyper and hypo DMRs

The DMR length distributed from 50 bp to 5000 bp, and primarily focused on the 50 bp and 200 bp, the CG length was most often observed at 52 bp, while both CHG and CHH peaked near 53 bp or 200 bp (Fig. S7). The CG content in hybrid LDM tissue was distributed at different regions with diverse methylation levels on a minority of chromosomes (Fig. 2A), while the CHG and CHH presented several significant differences in methylation level over all chromosomes (Fig. S8). The most significant difference (*p* < 0.05) in hypermethylated (hyper) DMRs between the two goat species was a length of 507 bp of CG content in the CGI region on chromosome 13 (areaStat = 1291.31), followed by a length of 384 bp in the CHH repeat region on chromosome 1 (areaStat = 1288.92) (Fig. 2B and Table S3), suggesting the maximal difference of hyper DMRs was located within regulatory regions. However, the most significant difference in hypomethylated (hypo) DMRs was a 655 bp region of CHH content in intron on chromosome 19 (areaStat = -3134.54), demonstrating the hypo DMRs mainly regulated the gene body region (Fig. 2B and Table S3).

Moreover, the DMRs represented a total of 3,269 annotated genes, including 1,586 genes in hyper DMRs, while 2566 genes in hypo DMRs, and 883 genes appeared in both DMRs (Fig. S9). The hyper DMRs in the CG context contained more genes than that of hypo DMRs in the promoter and 5’UTR regions (Fig. 2C), with fewer in the exon, intron, and 3’UTR, indicating the key role of hyper DMRs on the upstream regulatory region, while hypo DMRs predominantly functioned in the gene body and downstream (Fig. 2C). Overall, the hyper DMRs accounted for 38.26% of the gene body, promoter, 3’UTR and 5’UTR regions, whereas hypo DMRs occupied the remaining 61.74% (Fig. 2D), suggesting the total number of hypo DMR genes in the gene body and regulatory regions was significantly higher than those of hyper DMRs. However, the areaStat of the most significantly different genes in hyper DMRs was significantly elevated compared to hypo DMRs (Fig. 2D).

### The enriched pathways of DMRs related with muscle growth in LDM tissues

**Fig. 3:**
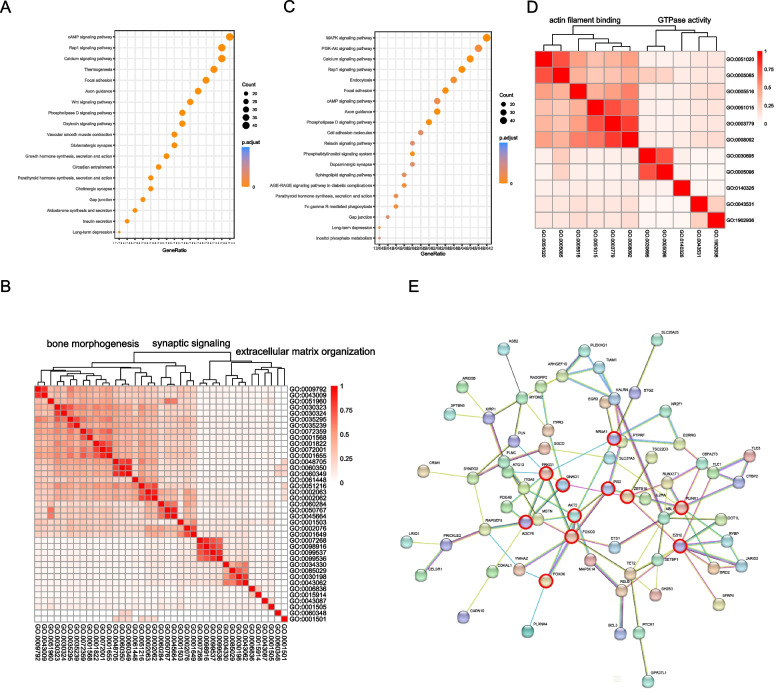
Functional analysis of the gene sets within DMRs and DEGs. The **A** KEGG and **B** GO enrichment of hyper DMR genes. **C** KEGG and **D** GO enrichment of hypo DMR genes. **E** PPI network of genes intersecting between DMRs and DEGs. The 11 hub genes are highlighted by a red outline

The genes of hyper DMRs in LDM tissue were enriched in the Rap1 signaling pathway and calcium signaling pathway (Fig. 3A). Moreover, the biological processes from GO analysis were separated into three categories: bone morphogenesis, synaptic signaling, and extracellular matrix organization (Fig. 3B), All of these impact myofiber formation and differentiation. Importantly, bone morphogenesis is closely tied to skeletal muscle growth as it took up the largest proportion of GO terms. Additionally, genes of hypo DMRs were primarily enriched in the mitogen-activated protein kinase (MAPK) signaling pathway, Phosphatidylinositol-3-kinase (PI3K-Akt) signaling pathway, calcium signaling pathway (Fig. 3C), and the molecular function of actin filament binding and GTPase activity (Fig. 3D), which played an important role in regulating myofiber activity and energy metabolism. Notably, Adenylate Cyclase 5 (*ADCY5*) and AKT Serine/Threonine Kinase 2 (*AKT2*) were involved in almost all the top pathways of hyper DMRs (Tables S4 and S5). AKT2 was considered an essential kinase for most vital activities, including cellular proliferation and differentiation, translation, and protein synthesis. Thus, the AKT2 would cause nutritional deficiency, inflammation, and immune dysfunction across tissues.

Furthermore, the DEGs intersected with the genes of hyper and hypo DMRs, and a gene set of 100 DEGs was obtained within hyper DMRs and 89 DEGs within hypo DMRs, respectively, resulting in 166 unique DMR genes (Table S6). Notably, the PPI network constructed by the 166 genes identified 11 proteins with strong correlation with others (Fig. 3E and Table S7), including Protein Kinase CGMP-Dependent 1 (PRKG1), AKT2, Forkhead Box O3 (FOXO3), Forkhead Box O6 (FOXO6), G Protein Subunit Alpha O1 (GNAO1), ADCY5, Enhancer of Zeste 2 Polycomb Repressive Complex 2 Subunit (EZH2), Zinc Finger And BTB Domain Containing 16 (ZBTB16), Nuclear Receptor Subfamily 4 Group A Member 1 (NR4A1), Insulin Receptor Substrate 2 (IRS2) and RUNX Family Transcription Factor 1 (RUNX1). The 11 proteins regulated diverse functions, including nutrient metabolism, growth hormone synthesis, inflammation, and cellular immune activity. For instance, ADCY5, AKT2, and IRS2 are important proteins in muscle growth hormone synthesis, lipolysis, and glycogen storage, while GNAO1 is involved in cholinergic synapse regulation, as well as FOXO3 and FOXO6, which regulates the balance of apoptosis and autophagy.

### The selection of DMR genes associated with muscle growth

**Fig. 4:**
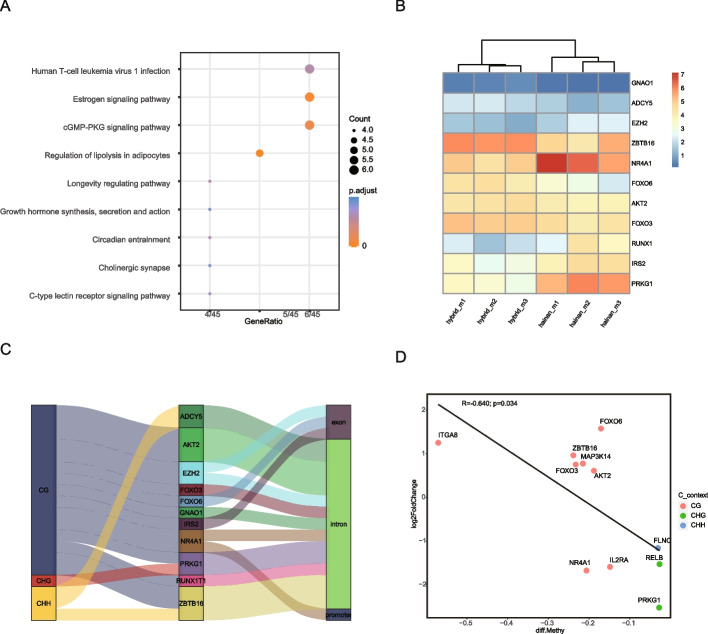
Functional analysis of gene sets intersecting between DMRs and DEGs. **A** KEGG enrichment of the intersection gene sets. **B** Expression patterns of the 11 hub genes from Hainan black goats and hybrid goats. **C** A depiction of the connections among genes, functional regions, and C contents of hub genes. **D** Correlations between differential gene expression and the degree of differential methylation levels within hypo DMRs

Furthermore, the gene sets intersecting with hyper DMRs and DEGs was enriched in nine pathways, focused on the regulation of lipolysis in adipocytes, cholinergic synapse, growth hormone synthesis, secretion and action (Fig. 4A), indicating the predominant function of energy metabolism within the LDM tissue. When comparing the pathways and the PPI network of hyper DMRs and DEGs, the critical genes that appeared across almost all the pathways also appeared in the PPI hub region, suggesting the consistency between the PPI and enriched pathways. The 11 hub genes were clearly separated into two clusters representing Hainan black goats and hybrid goats (Fig. 4B), with the methylation sites being dispersed across the gene body and regulatory regions, which contained all three contexts of CG, CHG and CHH (Fig. 4C, Table S8). Although it was clear that hypermethylation occurring in gene promoter regions would inhibit gene expression [27], the majority of the expression of 100 hyper DMR genes from the intersection DMRs and DEGs were not linked to their methylation level, nor related to gene promoter methylation (Fig. S10). However, 11 genes were identified with their expression significantly correlated with gene methylation levels in hypo DMRs (Fig. 4D, Table S9), and six genes (*AKT2*, *FOXO3, FOXO6, PRKG1, ZBTB16, NR4A1*) were also hub genes, functioning in myofiber growth, inflammation, and endocytosis.

### Correlations between gene expression and growth traits in the LEA

**Fig. 5:**
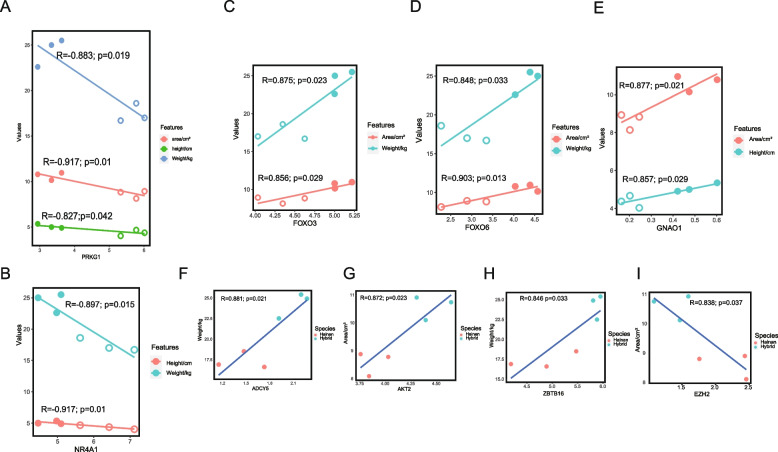
Correlations of hub genes with growth traits of LEA. **A***PRKG1* was negatively correlated with the weight, height, and LEA. **B***NR4A1* was negatively correlated with the height and weight. **C-E***FOXO3*, *FOXO6* and *GNAO1* were positively correlated with two of the three growth traits. **F-I***ADCY5*, *AKT2*, *ZBTB16* were positively correlated with one trait, and *EZH2* was negatively correlated with one trait. All hollow circles in **A-E** indicate Hainan black goats, and solid circles indicate hybrid goats

Three growth traits, including the area, height, and weight of the loin eye muscle area (LEA), were observed in both hybrid goats and Hainan black goats (Table S10). Correlations were established between genes expression and growth traits. *PRKG1* was the only gene exhibiting strongly negative correlations with all three growth traits (Fig. 5A), and *NR4A1* displayed the strongest negative correlation with two growth traits, namely height and weight (R = -0.91, P = 0.01; R = -0.89, P = 0.01) (Fig. 5B). Other genes, including *FOXO3*, *FOXO6*, and *GNAO1* were positively correlated with two growth traits of LEA (Fig. 5C-E). Specifically, *FOXO3* and *FOXO6* were both positively correlated with area and weight. Additionally, *ADCY5* was positively linked to weight (Fig. 5F), and participated in insulin-glucose signaling for glucose transferase [28]. *AKT2* gene accounted for the largest AKT isoform in human skeletal muscle [29] and was positively correlated with the LEA (Fig. 5G). Transcription factor *ZBTB16* and the target gene of transcription factor *EZH2* were linked to weight and LEA, respectively (Fig. 5H and I). Aside from two transcription factor-associated genes, the remaining genes in the PPI node scoring over 5 were *ADCY5*, *AKT2*, *PRKG1* and *FOXO3*, and the homologous genes of *FOXO3* and *FOXO6* frequently cooperated in glucose metabolism. Therefore, the five genes were considered to be the most critical genes for muscle fiber growth.

### The cooperation of critical genes in LDM

**Fig. 6:**
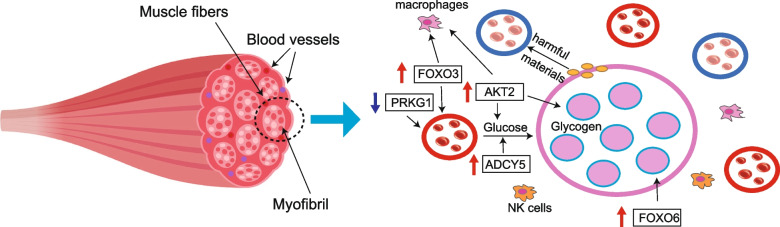
Identification and cooperation of hub genes associated with muscle growth. The left side depicts muscle fibers densely interspersed with blood vessels. The right side illustrates a microenvironment encompassing muscle fibers, blood vessels, and phagocytes. The central pink circle, filled with small myofibrils, represents a muscle fiber, surrounded by red and blue blood vessels, and several genes are interconnected around the muscle fiber, jointly contributing to glycogen storage. Red arrows indicate genes with increased expression, while blue arrows indicate genes with decreased expression

The muscle fibers were surrounded by numerous blood vessels and macrophages, constructing a microenvironment that ensured normal development and movement of myofibers. According to the enrichment pathways of DMR and DEGs, the protection of vascular endothelial cells may be critical for vasodilatation and the growth of LDM. The ADCY family, including *ADCY1*, *ADCY5*, *ADCY8*, and *ADCY9*, all took part in insulin secretion and growth hormone synthesis, secretion and action pathway, and vascular smooth muscle contraction upon DMR enrichment (Tables S4 and S5), especially *ADCY5* not only participated in these functions, also attributed to the regulation of glucose transport. *AKT2* was a hub gene linked with diverse signaling pathways, including phagocytosis and cellar growth (Tables S4 and S5). *PRKG1* and *FOXO3* were markers for the balance of autophagy of vascular endothelial cells [30, 31], while *PRKG1* also influenced the smooth vessel activation. Moreover, *FOXO6* maintained myotube proliferation and differentiation, and protected against atrophy [32]. Therefore, the healthy microenvironment for muscle growth was largely hinged on vascular activation and nutrient transport (Fig. 6).

The five most important genes, including *ADCY5*, *AKT2*, *FOXO3*, *FOXO6*, and *PRKG1*, were identified by RT-qPCR, and top four genes were significantly upregulated (*p* < 0.05) in hybrid goats compared to Hainan goats, while *PRKG1* was downregulated (*p* < 0.05) (Fig. S11). Therefore, the increasing expression of *ADCY5*, *AKT2*, *FOXO3* and *FOXO6* may be responsible for promoting muscle fiber growth. In addition, the decreasing expression of *PRKG1* suppressed vasoconstriction to release glucose more easily from the blood.

## Discussion

DNA methylation is an epigenetic regulation approach that is important for gene expression, tissue development [33], inflammation [34], autophagy and apoptosis [35]. The purpose of this study was to investigate the effects of DNA methylation on the LDM growth-related genes. Although the genome-wide methylation patterns of LDM between Hainan black goats and hybrid goats were generally similar (Fig. 1A and B), the gene number and functions were massively different during the DMR. The number of methylation sites in the gene body within hypo DMR was significantly higher than that within hyper DMR (Fig. 2C). In contrast, genes in hyper DMR also acted as hypermethylated genes with retrocopies available for glycan and lipid synthesis [36, 37]. The transcription factor *EZH2*, triggered DNA methylation in most cell activations [38], which were significantly downregulated expression in hybrid goats (*p* < 0.05), consisting of a negative correlation with LEA (Fig. 5I).

The DMR gene functions were focused on nutrient metabolism, growth hormone synthesis, vasodilatation, and endocytosis. Skeletal muscle is highly vascularized, and the muscle stem cell proliferation was accompanied by remarkable vascular and neuronal regeneration and differentiation [38, 39]. Vasodilatation could promote the skeletal muscle cells to uptake glucose [40]. Moreover, the inhibition of overexpressed *PRKG1* could limit vascular sclerosis to reverse aortopathy in Marfan syndrome mice [31], suggesting that *PRKG1* might be important in blood vessel activity. In this study, the expression of *PRKG1* was negatively correlated with the height and weight of LEA (Fig. 5A), which might be due to its decreasing expression benefitting vasodilatation to facilitate nutrient release, and acting as a switch to turn on glucose metabolism.

Similarly to previous studies, PI3K-Akt and MAPK signaling pathways activation could stimulate LDM development and differentiation in sheep and goats [41, 42], and these pathways were enriched in goat hypo DMR in this study. Notably, *FOXO3*, *FOXO6* and *AKT2* genes were related to PI3K-AKT signaling pathway (Table S5), which was important for skeletal muscle protein synthesis [43]. FOXO gene family is evolutionarily conserved, and *FOXO3* has been linked to longevity due to its vascular protection and regeneration [44]. Moreover, *FOXO3* acted as a cell surveillance mechanism to balance the apoptosis and autophagy [30], and their imbalance caused dystrophic features of skeletal muscle [45]. Furthermore, *FOXO3* eliminated senescent cells and harmful substances through hypo methylation [46]. Similarly, the homologous gene *FOXO6* maintained myotubes and protected against skeletal muscle atrophy [32]. In this study, both *FOXO3* and *FOXO6* were positively correlated with the area and weight of loin eye muscle, and both were located in hypo DMR. Therefore, it was clear that these two genes promoted myofiber development of skeletal muscle. In contrast, the methylation caused by maternal undernutrition treatment had no significant effects on the methylation of *PI3K* and *AKT* genes in goat semitendinosus (ST) and vastus lateralis (VL) muscles, nor did influence most of the gene expression levels of myogenic factors (MYFs) [47].

Besides the important genes in the top pathways, several genes were correlated to numerous functions and associated with the growth traits of muscle. For example, *NR4A1* was a glucose indicator as its expression was related to the insulin sensitivity of skeletal muscle [48], and its reduced methylation level influenced gene expression in adult sheep LDM [43]. Moreover, *IRS2* exhibited diverse functional features of insulin-dependent regulation of glucose and lipid metabolism in skeletal muscle and liver. Therefore, reduced expression of *NR4A1* and *IRS2* could inhibit sugar usage, whereas promote glycogen storage in LDM. Contrary to sheep containing opposite methylation levels and gene expression [43], these genes were downregulated (*p* < 0.05) in hybrid goats LDM compared to Hainan goats, indicating the different effects on gene expression caused by methylation in goats and sheep. Similarly, *ZBTB16* expression was accompanied by insulin-stimulated glucose transitioning into skeletal muscle glycogen, it acted as a surveillant for glucose quantity in muscles [49], therefore, the upregulated (*p* < 0.05) *ZBTB16* was response for muscular energy provision. *EZH2* was critical for the maintenance of smooth muscle physical form and function, and the inhibition of *EZH2* expression through promoter methylation could improve smooth muscle cell development [50]. However, its function in goat muscle was not reported. In this study, the downregulated (*p* < 0.05) *EZH2* gene may enhance the transport function of vascular smooth muscle in hybrid goats LDM. The missense variants of *GNAO1* present with paroxysmal dystonic manifestations, thus, this gene plays a role in muscular tension [51], and its increasing expression might enhance the LDM tension of hybrid goats. *RUNX1* contributed to myofiber development and differentiation, and induced muscle regeneration by promoting satellite cell proliferation [52], indicating the increased expression of *RUNX1* improved hybrid goats muscular growth.

Importantly, glucose transport and glycogen synthesis preferred to select *ADCY5*-mediated insulin-glucose pattern in LDM [53]. Glycogen storage was essential for myofiber movement [54], and the deletion of *ADCY5* was related to abnormal muscle activity during active movement as neurotransmitter disorders caused by glucose transporter deficiency [55]. In this study, upregulated (*p* < 0.05) *ADCY5* expression was positively correlated with the weight of LEA (Fig. 5F), which might be due to its linkage to glucose transport. The *AKT2* gene accounts for the largest percentage of AKT isoform in skeletal muscle to regulate myoblast differentiation and control the morphology of the muscle fibers [29], which may explain its positive correlation with the LEA. Further, other genes related to myofiber proliferation and differentiation also positively correlated with LEA and contributed to goat LDM growth.

## Conclusion

This study investigated the DNA methylation differences between the LDM of Hainan black goats and hybrid goats, acquired the critical genes, and analyzed their functions on myofiber growth. Notably, nine genes, including *PRKG1*, *AKT2*, *FOXO3*, *FOXO6*, *GNAO1*, *ADCY5*, *EZH2*, *ZBTB16*, and *NR4A1*, were significantly correlated (*p* < 0.05) with three growth traits of LDM partially or totally, and in particular, *PRKG1* was negatively related to all growth traits. The five key genes identified in DMR connected nutrient transport, vasoconstriction, and inflammation. Specifically, *FOXO3* and *FOXO6* monitored the balance of apoptosis and autophagy to maintain the immune microenvironment; *PRKG1* regulated the vasoconstriction to release nutrients, and its decreased expression promoted the glucose transfer and glycogen metabolism mediated by *ADCY5* and *AKT2* genes. Therefore, this study highlights the importance of methylation on the glycogen synthesis and myofiber growth-related genes in goat LDM, as well as provides the basic molecular mechanism for LDM development.

## Methods

### Tissue sampling

Given that the weight of hybrid goats (Nubian goats × Hainan black goats) was significantly higher than that of Hainan black goats (*p* < 0.05), we investigated the molecular mechanism ofzLDM growth, and examined the correlation between critical genes and growth traits of weight and LEA. The 12 individuals of 7-months-old male goats were purchased from Hainan Chengmai Fuyang Animal Husbandry Company (Hainan, China), and theses goats were maintained in a similar environment of free food and water for an adaptive week to reduce stress before slaughtering. There were 6 individuals for each group of Hainan black goats and hybrid goats, after the transitional period, all the individuals were experienced 24h of feed withdrawal, and three individuals for each group were randomly selected and transferred to the slaughterhouse of the HAAS YongFa goat experimental facility. The goats were stunned by electric shock, and exsanguinated, flushed and split at unconsciousness. All methods are reported in accordance with ARRIVE guidelines (https://arriveguidelines.org) for the reporting of animal experiments. The LDM were collected from each goat after slaughtering, and stored in liquid nitrogen for further analysis.

### Whole genome bisulfite sequencing and quality control

The DNA was extracted from LDM tissues by DNA extraction kit (Qiagen, 69504), and the concentration was detected by Nanodrop and Qubit 4.0, and then the high-quality DNA was sent to Novogene Company and subjected to WGBS, which was treated by bisulfite to separate methylated and unmethylated base C firstly, and then performed library construction of PE150 [56]. The inserted size of library was verified using an Agilent DNA-1000 Kit on an Agilent 2100 Bioanalyzer (Agilent Technologies, Santa Clara, CA, USA), and each library was sequencing on the Illumina NovaSeq 6000 platform. The raw sequenced data was evaluated by FastQC, and trimmed by fastp [57].

### Methylation stat analysis

The clean data was mapped to goat genome reference (ARS1, ASM170441v1) by Bismark [58], which transformed both the sequenced reads and genomic data from C to T or from G to A, separately, and four transforms were compared in pairs and removed duplications, and the mapping stats, containing unique mapping rate, sequencing depth and coverage, and the coverage of C, were evaluated to determine the high-quality data.

The CpG, CHG and CHH are three sequence contexts of methylated C (mC), where H is indicated as A, T, or C. The unique high quality bisulfite reads are classified into 6 types, which are methylated/unmethylated C in CpG, CHG, and CHH, separately. The accuracy of methylation detection is evaluated by binomial distribution [59], and the qualified methylation is required by sequencing depth more than 5 and q-value≤0.01. The methylation stats were summarized based on the genomic functional regions (promoter, exon, intron, CpG island (CGI)), where promoter was defined as the region of 2Kb upstream the gene transcription start site (TSS), and CGI and CGI shore were predicted by the cpgIslandExt (src/utils/cpgIslandExt/). DMR resulted from hybrid goats compared with Hainan black goats were identified by DSS package [60], taking account of main three factors, that are spatial correlation, sequencing depth of the sites, and the variance among biological replicates. The circos figures displayed methylation level, density and DMR were draw by TBtools [61].

### Expression of methylated genes

The RNA-seq data for each group were replicated for 3 individuals. Raw reads of RNA-seq were filtered by Trimmomatic (Version 0.36.6) [62] to removed adapters and low-quality reads, and clean reads were mapped to the goat genome reference (ARS1, ASM170441v1) using HISAT2 with default parameters (Version 2.2.1) [63], and read counts were calculated by HTSeq v. 0.9.1 [64]. Differentially expressed genes (DEGs) were identified by DEseq2 [65], requiring |log2(foldchange) |≥1, and adjusted by *p* ≤ 0.05. The primers sequences for RT-qPCR were designed and listed (Table S11).

### Enrichment analysis of methylation

The structure and gene annotation of DMR were further enriched for functions. The pathway analysis and functional classification were conducted by R package clusterProfiler [66] based on KEGG [67] and GO database, and the results were statistically adjusted by *p* ≤ 0.05. To simplify enriched GO terms, GOSemSim was used to calculate the similarity of GO terms and remove those redundancy terms, and only keep representative GO terms [68]. A protein-protein interactions (PPIs) network was constructed using the intersecting genes from DMRs and DEGs by STRING (https://cn.string-db.org). Subsequently, the visualization of enrichment results was implemented by R software.

### Statistical analysis

Both the correlation coefficient (R) either between methylation level and the gene expression, or between the gene expression and LDM traits, were calculated by R packages and detected by statistical method of pearson test, and the results were statistically adjusted by *p*≤0.05.

The statistical analysis for RT-qPCR was using Student's t test method within two-sided for 3 replicated individuals, and adjusted by *p* ≤ 0.05.

### Supplementary Information

**Additional file 1: Table S1. **The quality control of sequencing data. **Table S2. **The mapping rate of clean data of LDM tissues. **Table S3.** The distribution and the conditions of the top DMR between two goat species. **Table S4.** The enriched top pathways of hyper DMR. **Table S5.** The enriched top pathways of hypo DMR. **Table S6.** The intersection set of DMR genes and DEGs. **Table S7**. The PPI node score of 11 hub genes. **Table S8. **The methylation sites of 11 hub genes. **Table S9. **The expressions and methylation levels of 11 genes with correlations in hypo DMRs. **Table S10.** The growth traits of hybrid goats and Hainan black goats in LEA. **Table S11.** The primer sequences for RT-qPCR. **Fig. S1.** The sequencing depth distribution of all bases for Hainan black goats and hybrid goats. **Fig. S2.** The sequencing depth distribution of base accumulative in Hainan black goats and hybrid goats. The x-axis meant the sequencing depth; the y-axis meant the percentage of accumulative fraction of bases. **Fig. S3.** The average CG context number of chromosomes in Hainan black goats and hybrid goats. Blue color meant Hainan black goats, and orange color meant Hybrid goats. **Fig. S4.** The classification of mean mC proportions in LDM. **Fig. S5.** The methylation level distribution of mC contexts. **Fig. S6. **The average mC levels in different functional regions. **Fig. S7.** The length density distribution of three mC contexts in LDM. **Fig. S8.** The DMR analysis of LDM between Hainan black goats and hybrid goats. **Fig. S9.** The number of annotated genes in DMRs. **Fig. S10.** The correlations between methylation levels and the expression of DEGs in hyper DMR. **Fig. S11. **The RT-qPCR results of 5 most important genes.

## Data Availability

The raw data that support the findings of this study are available from the accession number PRJNA924997 in the NCBI BioProject database (https://www.ncbi.nlm.nih.gov/bioproject/?term=PRJNA924997).

## Abbreviations

LDM: longissimus dorsi muscle
WGBS: whole-genome bisulfite sequencing
DMRs: differentially methylated regions
DEGs: differentially expressed genes
MAPK: mitogen-activated protein kinase
PI3K-Akt: Phosphatidylinositol-3-kinase
*ADCY5*: adenylate cyclase 5
*AKT2*: AKT serine/threonine Kinase 2
*PRKG1*: protein kinase CGMP-dependent 1
*FOXO3*: forkhead box O3
*FOXO6*: forkhead box O6
*GNAO1*: G protein subunit alpha O1
*EZH2*: enhancer Of zeste 2 polycomb repressive complex 2 subunit
*ZBTB16*: zinc finger and BTB domain containing 16
*NR4A1*: nuclear receptor subfamily 4 group A member 1
*IRS2*: insulin receptor substrate 2
*RUNX1*: RUNX family transcription factor 1
LEA: loin eye muscle area
mC: methylated C
CGI: CpG island
TSS: transcription start site
PPIs: protein-protein interactions
