# Supplementary material for: Diverse WGBS profiles of longissimus dorsi muscle in Hainan black goats and hybrid goats
Source: BMC Genom Data. 2023 Dec 14;24:77. doi: 10.1186/s12863-023-01182-x (PMC10720224; doi:10.1186/s12863-023-01182-x)
Supplement: Supplementary file 1 — Additional file 1: Table S1. The quality control of sequencing data. Table S2. The mapping rate of clean data of LDM tissues. Table S3. The distribution and the conditions of the top DMR between two goat species. Table S4. The enriched top pathways of hyper DMR. Table S5. The enriched top pathways of hypo DMR. Table S6. The intersection set of DMR genes and DEGs. Table S7. The PPI node score of 11 hub genes. Table S8. The methylation sites of 11 hub genes. Table S9. The expressions and methylation levels of 11 genes with correlations in hypo DMRs. Table S10. The growth traits of hybrid goats and Hainan black goats in LEA. Table S11. The primer sequences for RT-qPCR. Fig. S1. The sequencing depth distribution of all bases for Hainan black goats and hybrid goats. Fig. S2. The sequencing depth distribution of base accumulative in Hainan black goats and hybrid goats. The x-axis meant the sequencing depth; the y-axis meant the percentage of accumulative fraction of bases. Fig. S3. The average CG context number of chromosomes in Hainan black goats and hybrid goats. Blue color meant Hainan black goats, and orange color meant Hybrid goats. Fig. S4. The classification of mean mC proportions in LDM. Fig. S5. The methylation level distribution of mC contexts. Fig. S6. The average mC levels in different functional regions. Fig. S7. The length density distribution of three mC contexts in LDM. Fig. S8. The DMR analysis of LDM between Hainan black goats and hybrid goats. Fig. S9. The number of annotated genes in DMRs. Fig. S10. The correlations between methylation levels and the expression of DEGs in hyper DMR. Fig. S11. The RT-qPCR results of 5 most important genes. [file 12863_2023_1182_MOESM1_ESM.docx]

**Supplementary materials**

**Supplementary tables**

**Table S1** The quality control of sequencing data

| Sample name | Raw_  reads | Raw_bases (G) | clean_  reads | clean_bases  (G) | Clean_  Ratio (%) | Q20  (%) | Q30  (%) | GC  (%) | BS conversion rate (%) |
| --- | --- | --- | --- | --- | --- | --- | --- | --- | --- |
| hainan_m1 | 297475877 | 89.24 | 287667226 | 77.90 | 87.29 | 96.21 | 89.52 | 22.39 | 99.736 |
| hainan_m2 | 315768224 | 94.73 | 297555010 | 80.21 | 84.67 | 95.91 | 88.85 | 22.35 | 99.730 |
| hainan_m3 | 314301487 | 94.29 | 304536577 | 82.55 | 87.55 | 96.55 | 90.27 | 22.41 | 99.743 |
| hybrid_m1 | 305680901 | 91.70 | 295446298 | 79.90 | 87.13 | 96.09 | 89.20 | 22.42 | 99.741 |
| hybrid_m2 | 306786150 | 92.04 | 296422293 | 80.19 | 87.13 | 96.11 | 89.08 | 22.21 | 99.738 |
| hybrid_m3 | 304028922 | 91.21 | 294570048 | 79.86 | 87.56 | 96.34 | 89.63 | 22.40 | 99.738 |

(1) Clean Reads：The number of raw reads after trimming;

(2) Clean Bases(G)：The bases of clean reads, and the unit is Gb;

(3) Clean Ratio(%)：The percentage of clean bases of the raw bases;
(4) Q20(%)：The percent of the base quality more than Q20, Q20 meant the error rate was 1%;

(5) Q30(%)：The percent of the base quality more than Q30, Q30 meant the error rate was 0.1%;
(6) GC Content(%)：The percentage of bases G and C in all the bases;

(7) BS conversion rate(%)：Bisulfite sequencing, meant the percentage of C change into T.

**Table S2** The mapping rate of clean data of LDM tissues

| Samples | Total reads | Mapped reads | Unique Mapping rate (%) | Duplication rate (%) |
| --- | --- | --- | --- | --- |
| Hainan_1 | 287667226 | 232463885 | 80.81 | 7.13 |
| Hainan_2 | 297555010 | 238103519 | 80.02 | 7.00 |
| Hainan_3 | 304536577 | 247496876 | 81.27 | 10.17 |
| hybrid_1 | 295446298 | 239193322 | 80.96 | 7.03 |
| hybrid_2 | 296422293 | 242028802 | 81.65 | 6.97 |
| hybrid_3 | 294570048 | 240840471 | 81.76 | 7.55 |

(1) Total reads: The number of clean reads;
(2) Mapped reads: The number of unique reads mapping to the reference genome sequence;

(3) Unique Mapping rate (%): The percentage of the number of mapped reads in the total reads;

(4) Duplication rate (%): The percentage of the number of duplicated reads in the total reads.

**Table S3** The distribution and the conditions of the top DMR between two goat species

| Chromosomes | Start | End | Length | Hybrid_ meanMethy | Hainan_ meanMethy | diff.  Methy | AreaStat | C_context |
| --- | --- | --- | --- | --- | --- | --- | --- | --- |
| NC_030820.1 | 81859659 | 81860165 | 507 | 0.873825 | 0.587282 | 0.286543 | 1291.308 | CG |
| NC_030808.1 | 12868 | 13251 | 384 | 0.080119 | 0.002686 | 0.077433 | 1288.918 | CHH |
| NC_030825.1 | 54818924 | 54819366 | 443 | 0.546038 | 0.296969 | 0.24907 | 1259.546 | CG |
| NC_030825.1 | 2562100 | 2562739 | 640 | 0.449253 | 0.28138 | 0.167873 | 1223.103 | CG |
| NC_030825.1 | 2562100 | 2562739 | 640 | 0.449253 | 0.28138 | 0.167873 | 1223.103 | CG |
| NC_030825.1 | 2562100 | 2562739 | 640 | 0.449253 | 0.28138 | 0.167873 | 1223.103 | CG |
| NC_030826.1 | 56271321 | 56271853 | 533 | 0.514142 | 0.283447 | 0.230696 | 1204.557 | CG |
| NC_030826.1 | 56271321 | 56271853 | 533 | 0.514142 | 0.283447 | 0.230696 | 1204.557 | CG |
| NC_030826.1 | 56271321 | 56271853 | 533 | 0.514142 | 0.283447 | 0.230696 | 1204.557 | CG |
| NC_030826.1 | 56271321 | 56271853 | 533 | 0.514142 | 0.283447 | 0.230696 | 1204.557 | CG |
| NC_030814.1 | 89297728 | 89298397 | 670 | 0.240003 | 0.111207 | 0.128796 | 1174.677 | CG |
| NC_030826.1 | 61683597 | 61684251 | 655 | 0.001671 | 0.060242 | -0.05857 | -3134.54 | CHH |
| NC_030833.1 | 42160949 | 42161460 | 512 | 0.002262 | 0.073491 | -0.07123 | -2460.76 | CHH |
| NC_030833.1 | 42160949 | 42161460 | 512 | 0.002262 | 0.073491 | -0.07123 | -2460.76 | CHH |
| NC_030833.1 | 42160949 | 42161460 | 512 | 0.002262 | 0.073491 | -0.07123 | -2460.76 | CHH |
| NC_030833.1 | 42160949 | 42161460 | 512 | 0.002262 | 0.073491 | -0.07123 | -2460.76 | CHH |
| NC_030833.1 | 42160949 | 42161460 | 512 | 0.002262 | 0.073491 | -0.07123 | -2460.76 | CHH |
| NC_030832.1 | 42502786 | 42504163 | 1378 | 0.328108 | 0.727248 | -0.39914 | -2449.93 | CG |
| NC_030832.1 | 42502786 | 42504163 | 1378 | 0.328108 | 0.727248 | -0.39914 | -2449.93 | CG |
| NC_030818.1 | 12932659 | 12932968 | 310 | 0.001655 | 0.123293 | -0.12164 | -2035.41 | CHH |
| NC_030818.1 | 12932659 | 12932968 | 310 | 0.001655 | 0.123293 | -0.12164 | -2035.41 | CHH |

Start and end meant the start site and end site of the DMR region; diff.Methy and areaStat were two indicators of the statistical differences of DMR. If the diff.Methy more than 0, it meant the methylation level of DMR region in hybrid goats was significantly higher than that of Hainan black goats, otherwise was significantly lower than that of Hainan goats. Absolute value of areaStat was used to estimate the degree of DMR.

**Table S4** The enriched top pathways of hyper DMR

| ID | Description | GeneRatio | BgRatio | *P* value | *P*.adjust | geneID | Count |
| --- | --- | --- | --- | --- | --- | --- | --- |
| chx04724 | Glutamatergic synapse | 28/708 | 111/8895 | 2.52E-08 | 4.16E-06 | GRIK1/ADCY5/ADCY1/GRIN2B/  SHANK3/CACNA1A/GRIK2/GNG2/  GRIN1/PLCB1/PLCB4/LOC108633189/  LOC102188874/ADCY8/SLC1A2/GRIA4/  GNAO1/GRIK5/PRKCA/GRM7/CACNA1D/  GRM4/ITPR3/ADCY9/GRIN2A/PRKCB/  SHANK2/GRIA3 | 28 |
| chx04713 | Circadian entrainment | 26/708 | 98/8895 | 2.60E-08 | 4.16E-06 | ADCY5/C3H1orf226/ADCY1/GRIN2B/  CACNA1I/CAMK2A/RPS6KA5/GNG2/  GRIN1/PLCB1/PLCB4/LOC108633189/  LOC102188874/ADCY8/GRIA4/NOS1/  GNAO1/RYR1/PRKCA/CACNA1D/ITPR3/  ADCY9/GRIN2A/PRKCB/PRKG1/GRIA3 | 26 |
| chx04015 | Rap1 signaling pathway | 41/708 | 211/8895 | 5.78E-08 | 6.16E-06 | TIAM1/ADCY5/RAPGEF4/ADCY1/MAGI2/  GRIN2B/PDGFB/EFNA5/VAV1/FLT4/AFDN/  TLN2/VAV2/GRIN1/PLCB1/PLCB4/PARD3/  LOC108633189/LOC102188874/SRC/PLCG1/  ADCY8/DRD2/RASSF5/GNAO1/CDH1/AKT2/  MAP2K3/PRKCA/LCP2/FGF18/PRKD1/EVL/  MAGI1/RAF1/ADCY9/GRIN2A/PRKCB/  PDGFA/SIPA1L2/FGF16 | 41 |
| chx04540 | Gap junction | 23/708 | 88/8895 | 2.23E-07 | 1.79E-05 | ADCY5/MAP3K2/ADCY1/PDGFB/GNA11/  MAP2K5/PLCB1/PLCB4/LOC108633189/  LOC102188874/SRC/ADCY8/DRD2/GRB2/  PRKCA/RAF1/ITPR3/ADCY9/PRKCB/PDGFA/  PRKG1/LOC102187765/LOC102173837 | 23 |
| chx04072 | Phospholipase D signaling pathway | 31/708 | 147/8895 | 3.78E-07 | 2.06E-05 | ADCY5/AGPAT3/RAPGEF4/RHEB/ADCY1/  DGKB/PDGFB/DGKQ/PIP5K1C/PIP5K1B/  SHC3/PLCB1/PLCB4/LOC108633189/  LOC102188874/PLCG1/ADCY8/DNM3/  PLCG2/AKT2/LOC102191173/GRB2/  PRKCA/GRM7/RAF1/GRM4/ADCY9/  CYTH3/GNA12/PDGFA/DGKK | 31 |
| chx04935 | Growth hormone synthesis, secretion  and action | 27/708 | 118/8895 | 3.87E-07 | 2.06E-05 | ADCY5/ADCY1/EP300/GNA11/SHC3/  IRS2/PLCB1/PLCB4/LOC108633189/  LOC102188874/PLCG1/ADCY8/PTK2/  CACNA1S/PLCG2/AKT2/MAP2K3/  STAT5B/GRB2/PRKCA/CACNA1D/  RAF1/ITPR3/IGFALS/ADCY9/PRKCB/IRS4 | 27 |
| chx04024 | cAMP signaling  pathway | 42/708 | 238/8895 | 6.40E-07 | 2.93E-05 | TIAM1/ADCY5/RAPGEF4/VIPR2/GLI3/  ADCY1/GRIN2B/EP300/CAMK4/CAMK2A/  VAV1/PTCH1/PDE10A/AFDN/VAV2/GRIN1/  LOC108637249/LOC108637252/  LOC108637253/LOC102173859/  LOC102181111/LOC102179751/  LOC108633189/LOC102188874/ADCY8/  DRD2/GRIA4/CACNA1S/AKT2/LIPE/  SOX9/PDE4D/OXTR/CACNA1D/RAF1/  GABBR1/NFATC1/ADCY9/GRIN2A/  CHRM1/GRIA3/ATP2B3 | 42 |
| chx04921 | Oxytocin signaling pathway | 31/708 | 153/8895 | 9.50E-07 | 3.80E-05 | ADCY5/PRKAG3/PRKAG2/ADCY1/CAMK4/  CAMK2A/NFATC4/MAP2K5/PLCB1/PLCB4/  LOC108633189/LOC102188874/SRC/ADCY8/  CACNA1S/PPP1R12B/CAMKK2/GNAO1/  RYR1/PRKCA/CACNG4/OXTR/CACNA2D3/  CACNA1D/RAF1/MYLK4/ITPR3/NFATC1/  ADCY9/PRKCB/CCND1 | 31 |
| chx04360 | Axon guidance | 34/708 | 180/8895 | 1.56E-06 | 5.33E-05 | ROBO1/EPHA3/EPHB1/EPHB2/EPHA4/  NTNG1/EPHB6/PLXNC1/UNC5C/BMPR1B/  ABLIM2/EFNA5/RASA1/CAMK2A/SEMA6B/  DPYSL2/PTCH1/RGS3/PAK6/NFATC4/GDF7/  PARD3/SRC/PLCG1/PTK2/PLCG2/PRKCA/  SLIT3/RGMA/SRGAP3/RAF1/LOC102173800/  CXCL12/L1CAM | 34 |
| chx04020 | Calcium signaling pathway | 41/708 | 238/8895 | 1.66E-06 | 5.33E-05 | ERBB4/PDE1A/ADCY1/PDGFB/CACNA1I/  STIM2/CAMK4/CAMK2A/GNA11/CACNA1A/  FLT4/ADRA1A/SLC8A3/CACNA1B/GRIN1/  PLCB1/PLCB4/LOC108633189/LOC102188874/  PLCG1/ADCY8/CACNA1S/NOS1/PLCG2/  RYR1/PRKCA/FGF18/NTRK3/OXTR/  CACNA1D/MYLK4/ITPR3/ADCY9/GRIN2A/  PRKCB/PDGFA/CHRM1/TPCN2/FGF16/  SLC25A5/ATP2B3 | 41 |
| chx04928 | Parathyroid hormone synthesis, secretion  and action | 24/708 | 106/8895 | 2.09E-06 | 6.07E-05 | ADCY5/ARHGEF11/ADCY1/SP1/LRP6/  GNA11/TNFSF11/PLCB1/PLCB4/  LOC108633189/LOC102188874/  LOC102178921/ADCY8/MMP17/  PRKCA/PDE4D/PTH1R/RAF1/ITPR3/  BCL2/ADCY9/PRKCB/GNA12/LRP5 | 24 |
| chx04730 | Long-term depression | 17/708 | 60/8895 | 2.54E-06 | 6.46E-05 | GNA11/CACNA1A/PLCB1/PLCB4/  LOC108633189/LOC102188874/NOS1/  GNAO1/RYR1/PRKCA/RAF1/ITPR3/  PRKCB/GNA12/PRKG1/GRIA3/GNAZ | 17 |
| chx04310 | Wnt signaling pathway | 32/708 | 168/8895 | 2.63E-06 | 6.46E-05 | WNT10A/VANGL2/CUL1/LRP6/CCND2/  EP300/WNT7B/CTBP1/CAMK2A/WNT9A/  NFATC4/SMAD3/APC/PLCB1/PLCB4/  RSPO2/LGR4/LGR6/WNT3/AXIN2/PRKCA/  RUVBL1/DAAM2/PPARD/NFATC1/PRKCB/  CTBP2/SFRP5/LRP5/CCND1/TBL1X/  LOC102180219 | 32 |
| chx04911 | Insulin secretion | 21/708 | 87/8895 | 3.05E-06 | 6.97E-05 | ADCY5/RAPGEF4/ADCY1/CAMK2A/  GNA11/KCNN1/PDX1/PLCB1/PLCB4/  LOC108633189/LOC102188874/ADCY8/  CACNA1S/KCNN4/PRKCA/CACNA1D/  ITPR3/ADCY9/PRKCB/KCNU1/KCNMA1 | 21 |
| chx04925 | Aldosterone synthesis  and secretion | 22/708 | 95/8895 | 3.73E-06 | 7.95E-05 | ADCY5/ADCY1/CACNA1I/CAMK4/  CAMK2A/GNA11/PLCB1/PLCB4/  LOC108633189/LOC102188874/ADCY8/  PDE2A/CACNA1S/LIPE/PRKCA/PRKD1/  CACNA1D/ITPR3/ADCY9/PRKCB/DAGLB/  ATP2B3 | 22 |
| chx04510 | Focal adhesion | 36/708 | 205/8895 | 4.57E-06 | 9.14E-05 | COL4A4/THBS3/VWF/CCND2/PDGFB/  PIP5K1C/VAV1/FLT4/PIP5K1B/SHC3/  TLN2/PAK6/VAV2/COL4A2/COL4A1/  SRC/PTK2/CAPN2/TNR/LAMC1/  PPP1R12B/AKT2/ARHGAP35/COL1A1/  GRB2/PRKCA/ITGA9/RAF1/MYLK4/  BCL2/PRKCB/PDGFA/DOCK1/CCND1/  LOC108634775/LOC108635476 | 36 |
| chx04725 | Cholinergic synapse | 24/708 | 111/8895 | 4.94E-06 | 9.30E-05 | ADCY5/ADCY1/CAMK4/CAMK2A/  GNA11/CACNA1A/GNG2/CACNA1B/  PLCB1/PLCB4/ADCY8/CACNA1S/  GNAO1/AKT2/PRKCA/CACNA1D/  ITPR3/BCL2/ADCY9/PRKCB/ACHE/  SLC18A3/CHRM1/KCNQ1 | 24 |
| chx04270 | Vascular smooth muscle contraction | 28/708 | 145/8895 | 8.41E-06 | 0.00015 | ADCY5/NPPC/RAMP1/ARHGEF11/  ADCY1/GNA11/ADRA1A/PLCB1/  PLCB4/LOC108633189/LOC102188874/  ADCY8/ADM/LOC102189885/CACNA1S/  PPP1R12B/PTGIR/PRKCA/CACNA1D/  RAF1/MYLK4/ITPR3/ADCY9/PRKCB/  GNA12/PRKG1/KCNU1/KCNMA1 | 28 |
| chx04714 | Thermogenesis | 38/708 | 237/8895 | 2.22E-05 | 0.000374 | ND1/ND2/COX1/COX2/ATP8/ATP6/  COX3/ND3/ND4/ND5/CYTB/ND6/  LOC102170266/ADCY5/  SDHB/PRKAG3/BMP8B/PRKAG2/  RHEB/ADCY1/PPARGC1A/ACSL6/  RPS6KA2/LOC108633189/  LOC102188874/ADCY8/PRDM16/  DPF1/LIPE/CPT1C/MAP2K3/GRB2/  SMARCC1/MGLL/ADCY9/PRKG1/  CPT1A/LOC102172862 | 38 |

The ID was the KEGG pathway ID; the description was the name of the pathway; the GeneRatio was the enriched gene number of corresponding pathway divided the total gene number of all enriched pathways; the BgRatio was the total gene number of corresponding pathway divided the total genes of all enriched pathways; *P* value was the statistical *P* value; *P*.adjust was the adjust *P* value; geneID was the enriched genes of corresponding pathway; Count was the enriched gene number of corresponding pathway.

**Table S5** The enriched top pathways of hypo DMR

| ID | Description | GeneRatio | BgRatio | *P* value | *P*.adjust | geneID | Count |
| --- | --- | --- | --- | --- | --- | --- | --- |
| chx04010 | MAPK signaling pathway | 48/642 | 295/8894 | 6.48E-08 | 1.01E-05 | EFNA4/FLNC/CACNA2D1/KITLG/  NR4A1/CACNB3/PTPRR/CACNG2/  DUSP16/FGF6/CACNA2D4/CACNA1C/  MAPK8IP2/MAPK10/EFNA5/EFNA2/  INSR/MAP3K4/RPS6KA2/RPS6KA5/  MAP2K5/TGFA/CACNA1B/FLT1/  TRAF6/MAPKAPK2/LOC102184834/  CACNA1S/PTPN7/TAOK3/PDGFC/  RAPGEF2/AKT2/RELB/MAP3K14/  MAPT/PRKCA/CACNG5/IGF1R/AKT1/  CACNA2D2/VEGFA/MAPK8IP3/PRKCB/  IKBKB/RASGRP2/IGF2/LOC102179926 | 48 |
| chx04015 | Rap1 signaling pathway | 38/642 | 211/8894 | 1.10E-07 | 1.14E-05 | ITGB2/RAPGEF4/VAV3/MAGI3/EFNA4/  MAGI2/KITLG/RAPGEF3/FGF6/EFNA5/  EFNA2/VAV1/INSR/F2RL3/LPAR1/AFDN/  PLCB2/RALGDS/VAV2/FLT1/PLCB4/PARD3/  LOC102188874/LOC108633189/PDGFC/  RAPGEF2/AKT2/PRKCA/IGF1R/PRKD1/  AKT1/MAGI1/VEGFA/GRIN2A/PRKCB/  RASGRP2/SIPA1/ADORA2A | 38 |
| chx04020 | Calcium signaling pathway | 40/642 | 238/8894 | 3.50E-07 | 2.72E-05 | HTR6/PDE1A/CASQ2/NOS3/ITPR2/PLCZ1/  FGF6/CACNA1C/CAMK4/GNA15/PLN/SLC8A3/  ITPKA/PLCB2/RYR3/SLC8A1/CACNA1B/FLT1/  PLCB4/ADRA1D/LOC102188874/LOC108633189/  TRHR/ASPH/P2RX3/ITPKB/LOC102184834/  CACNA1S/ATP2A2/PDGFC/PRKCA/VEGFA/  GRIN2A/PRKCB/MCU/RET/GRM5/TPCN2/  ADORA2A/ATP2B3 | 40 |
| chx04072 | Phospholipase D signaling pathway | 29/642 | 147/8894 | 5.11E-07 | 3.18E-05 | AGPAT3/RAPGEF4/DGKD/DGKB/KITLG/  RAPGEF3/CYTH4/INSR/ARF1/GRM6/  PIP5K1B/LPAR1/PLCB2/RALGDS/AGPAT2/  PLCB4/LOC102188874/LOC108633189/  DGKZ/DNM3/PDGFC/AKT2/PLD2/CYTH1/  PRKCA/AKT1/GRM7/CYTH3/GRM5 | 29 |
| chx04360 | Axon guidance | 32/642 | 180/8894 | 1.51E-06 | 7.83E-05 | ROBO1/EPHA3/EPHA8/EPHA4/NGEF/  EFNA4/EPHB6/PLXNA4/SRGAP1/  EPHA5/ABLIM2/EFNA5/EFNA2/UNC5A/  SEMA6A/RGS3/EPHA7/NCK2/ABL1/  PARD3/BMP7/ILK/SRGAP2/PLXNA2/  PRKCA/SLIT3/SEMA3B/PLXNB1/PDPK1/  EPHB4/UNC5D/LOC102177760 | 32 |
| chx04510 | Focal adhesion | 33/642 | 205/8894 | 9.72E-06 | 0.000432 | COL4A3/COL4A4/VAV3/FLNC/VWF/  PARVB/MAPK10/VAV1/PIP5K1B/  THBS2/ACTN1/VAV2/COL4A2/  COL4A1/FLT1/ITGA8/ILK/PARVA/  LAMB3/PPP1R12B/PXN/PDGFC/AKT2/  PRKCA/IGF1R/AKT1/VEGFA/LAMA3/  BCL2/PDPK1/PRKCB/DOCK1/LOC108634846 | 33 |
| chx04070 | Phosphatidylinositol signaling system | 20/642 | 97/8894 | 1.52E-05 | 0.000589 | DGKD/INPP5D/DGKB/ITPR2/PLCZ1/  PIK3C2G/CDS1/PIP5K1B/SYNJ2/  ITPKA/PLCB2/PLCB4/DGKZ/ITPKB/  PRKCA/MTMR14/PRKCB/INPP5A/  MTMR7/OCRL | 20 |
| chx04144 | Endocytosis | 35/642 | 255/8894 | 0.000161 | 0.004382 | GRK7/ARPC2/BIN1/AGAP1/AGAP3/  CYTH4/GRK4/PSD2/EPS15L1/ARF1/  PIP5K1B/IGF2R/MVB12B/IL2RA/  PARD3/ITCH/ASAP1/SMAP1/TRAF6/  DNM3/AP2B1/PLD2/CYTH1/IGF1R/  IQSEC1/LOC108633505/LOC102188814/  LOC102168905/LOC102170045/  LOC102185917/SMAD2/NEDD4L/  RAB11FIP3/CYTH3/PSD3 | 35 |
| chx04666 | Fc gamma R-mediated phagocytosis | 18/642 | 97/8894 | 0.000169 | 0.004382 | ARPC2/BIN1/INPP5D/VAV3/PLA2G6/  VAV1/PIP5K1B/GSN/VAV2/ASAP1/  PTPRC/LOC108637962/AKT2/PLD2/  PRKCA/AKT1/PRKCB/DOCK1 | 18 |
| chx04933 | AGE-RAGE signaling pathway in diabetic complications | 19/642 | 107/8894 | 0.000206 | 0.004919 | COL4A3/COL4A4/NOS3/CDKN1B/  MAPK10/PLCB2/COL4A2/COL4A1/  PLCB4/AKT2/BAX/PRKCA/AKT1/  VEGFA/SMAD2/BCL2/PRKCB/  LOC108634845/LOC108634846 | 19 |
| chx04730 | Long-term depression | 13/642 | 60/8894 | 0.000279 | 0.00578 | ITPR2/GRID2/PRKG2/PLCB2/PLCB4/  LOC102188874/LOC108633189/  LOC108637886/PRKCA/IGF1R/PRKCB/  PRKG1/GNAZ | 13 |
| chx04151 | PI3K-Akt signaling pathway | 45/642 | 369/8894 | 0.000325 | 0.006325 | COL4A3/COL4A4/EFNA4/NOS3/MAGI2/  CDK6/KITLG/NR4A1/CDKN1B/VWF/  FGF6/PPP2R2C/EFNA5/EFNA2/GNG7/  INSR/LPAR1/FOXO3/THBS2/TGFA/  COL4A2/COL4A1/FLT1/IL2RA/ITGA8/  YWHAZ/CREB3L1/LAMB3/LOC108637886/  PDGFC/AKT2/PRKCA/IGF1R/AKT1/MAGI1/  VEGFA/LAMA3/PHLPP1/BCL2/PDPK1/  IKBKB/IGF2/PPP2R3B/LOC108634606/  LOC108634846 | 45 |
| chx04024 | cAMP signaling pathway | 32/642 | 238/8894 | 0.000442 | 0.008079 | HTR6/RAPGEF4/PDE4B/VAV3/VIPR2/  RAPGEF3/CACNA1C/MAPK10/CAMK4/  AMH/VAV1/PLN/PDE10A/AFDN/CNGA3/  VAV2/LOC108637253/LOC102181111/  LOC102188874/LOC108633189/CREB3L1/  CACNA1S/ATP2A2/CNGB1/AKT2/PLD2/  SOX9/PDE4D/AKT1/GRIN2A/ADORA2A/  ATP2B3 | 32 |
| chx04928 | Parathyroid hormone synthesis, secretion and action | 18/642 | 106/8894 | 0.000527 | 0.00911 | PDE4B/ITPR2/SLC34A2/PLCB2/TNFSF11/  PLCB4/LOC102188874/LOC108633189/  MMP16/CREB3L1/PLD2/PRKCA/PDE4D/  AKAP13/BCL2/MMP25/PRKCB/LOC108634606 | 18 |
| chx04071 | Sphingolipid signaling pathway | 19/642 | 120/8894 | 0.000911 | 0.013496 | NOS3/MAPK10/PPP2R2C/ACER1/PLCB2/  PLCB4/SPTLC3/LOC108637886/AKT2/  BAX/PLD2/PRKCA/AKT1/BCL2/PDPK1/  ABCC1/PRKCB/SGMS1/PPP2R3B | 19 |
| chx04514 | Cell adhesion molecules | 23/642 | 160/8894 | 0.001105 | 0.014947 | NCAM2/ITGB2/PTPRF/NEGR1/CNTNAP2/  NRXN3/CD276/ITGA8/CDH4/SDC2/NCAM1/  CNTN2/CD34/PTPRC/NECTIN2/LRRC4B/  CLDN7/LOC108633505/LOC102188814/  LOC102168905/LOC102170045/LOC102185917/  CD226 | 23 |
| chx04728 | Dopaminergic synapse | 20/642 | 133/8894 | 0.001307 | 0.01602 | DRD3/ITPR2/CACNA1C/MAPK10/PPP2R2C/  GNG7/PLCB2/CACNA1B/PLCB4/LOC102188874/  LOC108633189/CREB3L1/LOC108637886/AKT2/  PRKCA/AKT1/GRIN2A/PRKCB/PPP2R3B/COMT | 20 |
| chx04926 | Relaxin signaling pathway | 20/642 | 134/8894 | 0.001435 | 0.01602 | COL4A3/COL4A4/NOS3/MAPK10/GNG7/GNA15/  PLCB2/COL4A2/COL4A1/PLCB4/LOC102188874/  LOC108633189/CREB3L1/AKT2/PRKCA/AKT1/  VEGFA/SMAD2/LOC108634845/LOC108634846 | 20 |
| chx04540 | Gap junction | 15/642 | 88/8894 | 0.001442 | 0.01602 | ITPR2/PRKG2/LPAR1/PLCB2/MAP2K5/PLCB4/  LOC102188874/LOC108633189/PDGFC/CSNK1D/  PRKCA/LOC102172952/PRKCB/PRKG1/GRM5 | 15 |
| chx00562 | Inositol phosphate metabolism | 13/642 | 72/8894 | 0.001718 | 0.018422 | INPP5D/PLCZ1/PIK3C2G/PIP5K1B/SYNJ2/ITPKA/  PLCB2/PLCB4/ITPKB/MTMR14/INPP5A/MTMR7/  OCRL | 13 |

The ID was the KEGG pathway ID; the description was the name of the pathway; the GeneRatio was the enriched gene number of corresponding pathway divided the total gene number of all enriched pathways; the BgRatio was the total gene number of corresponding pathway divided the total genes of all enriched pathways; *P* value was the statistical *P* value; *P*.adjust was the adjust *P* value; geneID was the enriched genes of corresponding pathway; Count was the enriched gene number of corresponding pathway.

**Table S6** The intersection set of DMR genes and DEGs

| ID | Symbols | baseMean | log2FoldChange | *P* value | padj | methyStat |
| --- | --- | --- | --- | --- | --- | --- |
| 1 | TIAM1 | 206.49 | -1.765 | 0.000461 | 0.01253 | hyper |
| 2 | ADCY5 | 279.404 | 0.934 | 0.001774 | 0.032915 | hyper |
| 3 | RUNX1 | 240.994 | -2.001 | 0.000106 | 0.004151 | hyper |
| 4 | GALNT15 | 201.472 | 2.403 | 7.53E-07 | 7.12E-05 | hyper |
| 5 | KIF17 | 130.196 | -2.025 | 0.001597 | 0.030424 | hyper/hypo |
| 6 | CLIC4 | 12096.185 | -0.786 | 0.000714 | 0.017181 | hyper |
| 7 | AHDC1 | 1350.898 | 0.965 | 0.000428 | 0.011843 | hyper |
| 8 | TNS1 | 8555.903 | 0.761 | 0.000884 | 0.019839 | hyper/hypo |
| 9 | RAPGEF4 | 236.933 | -1.021 | 0.001916 | 0.034421 | hyper/hypo |
| 10 | ZNF385B | 186.343 | -1.851 | 1.17E-05 | 0.000684 | hyper |
| 11 | LIMS2 | 967.267 | 0.794 | 3.58E-06 | 0.000281 | hyper |
| 12 | CAPN10 | 124.858 | 1.192 | 8.17E-06 | 0.00052 | hyper/hypo |
| 13 | TRABD2B | 68.65 | 1.28 | 0.000627 | 0.01582 | hyper/hypo |
| 14 | GIPC2 | 75.26 | -3.068 | 6.18E-06 | 0.000432 | hyper |
| 15 | EZH2 | 116.188 | -1.201 | 0.001566 | 0.030085 | hyper |
| 16 | SLC37A3 | 432.596 | -0.836 | 0.001274 | 0.025715 | hyper |
| 17 | PAWR | 220.742 | 1.181 | 0.001118 | 0.023534 | hyper |
| 18 | CELSR1 | 112.826 | 1.087 | 0.000806 | 0.018533 | hyper/hypo |
| 19 | SGMS2 | 484.378 | -2.673 | 1.85E-09 | 3.86E-07 | hyper |
| 20 | LNX1 | 427.256 | 1.424 | 0.001246 | 0.025352 | hyper/hypo |
| 21 | SORCS2 | 53.701 | 1.317 | 0.002567 | 0.042169 | hyper/hypo |
| 22 | C6H4orf48 | 146.451 | 1.04 | 0.002363 | 0.039964 | hyper/hypo |
| 23 | NAT8L | 25.053 | 1.962 | 0.003017 | 0.047182 | hyper/hypo |
| 24 | NR2F1 | 112.199 | 2.364 | 0.000235 | 0.007515 | hyper/hypo |
| 25 | MIDN | 3630.217 | -2.102 | 6.06E-13 | 2.84E-10 | hyper |
| 26 | ADAMTS10 | 226.414 | 1.1 | 0.002216 | 0.038384 | hyper |
| 27 | LRRC8E | 28.827 | -1.99 | 1.54E-05 | 0.000865 | hyper |
| 28 | MYO9B | 845.115 | -0.889 | 9.62E-05 | 0.003885 | hyper |
| 29 | PTCH1 | 124.959 | 1.606 | 3.30E-05 | 0.001648 | hyper |
| 30 | RGS3 | 736.193 | 0.872 | 0.001625 | 0.030809 | hyper/hypo |
| 31 | PAK6 | 31.75 | -2.936 | 2.16E-05 | 0.001151 | hyper |
| 32 | SH3RF3 | 105.71 | 1.008 | 0.000193 | 0.006501 | hyper |
| 33 | IRS2 | 1445.927 | -1.422 | 1.13E-05 | 0.000663 | hyper |
| 34 | FOXS1 | 286.187 | 1.674 | 3.03E-08 | 4.29E-06 | hyper |
| 35 | ADIG | 57.067 | 1.28 | 0.000929 | 0.020595 | hyper |
| 36 | JPH2 | 3272.723 | -0.657 | 0.000191 | 0.006472 | hyper/hypo |
| 37 | RUNX1T1 | 300.086 | 0.64 | 0.001366 | 0.027031 | hyper |
| 38 | EXT1 | 766.145 | -1.139 | 0.003031 | 0.047306 | hyper |
| 39 | ATG13 | 716.733 | 0.678 | 0.000607 | 0.01544 | hyper |
| 40 | LGR4 | 687.971 | -0.48 | 0.003188 | 0.048737 | hyper |
| 41 | ZBTB16 | 5956.002 | 0.963 | 0.002785 | 0.044916 | hyper/hypo |
| 42 | BTG2 | 11510.128 | -3.887 | 4.67E-23 | 8.76E-20 | hyper |
| 43 | ESRRG | 95.636 | 1.391 | 0.001006 | 0.021858 | hyper |
| 44 | TMEM201 | 543.389 | 1.226 | 1.81E-06 | 0.000155 | hyper |
| 45 | NCF2 | 127.201 | -2.735 | 3.59E-05 | 0.001757 | hyper |
| 46 | GPR37L1 | 40.481 | 3.387 | 0.000708 | 0.017163 | hyper |
| 47 | MN1 | 2250.437 | 1.092 | 0.001815 | 0.033336 | hyper |
| 48 | TMEM120B | 580.099 | 1.602 | 3.14E-10 | 7.86E-08 | hyper |
| 49 | RFLNA | 320.713 | 2.025 | 0.000207 | 0.006815 | hyper |
| 50 | TRIM2 | 764.955 | 1.118 | 7.07E-06 | 0.000472 | hyper |
| 51 | IL34 | 124.955 | 1.318 | 2.10E-06 | 0.000177 | hyper |
| 52 | COTL1 | 1127.043 | -0.953 | 0.000912 | 0.020383 | hyper |
| 53 | C18H16orf87 | 123.708 | -0.897 | 0.000586 | 0.015046 | hyper |
| 54 | GNAO1 | 39.764 | 1.45 | 0.000714 | 0.017181 | hyper |
| 55 | AKT2 | 1520.836 | 0.609 | 0.00118 | 0.024355 | hyper/hypo |
| 56 | BCL3 | 739.12 | -1.894 | 6.88E-05 | 0.002969 | hyper |
| 57 | PEG3 | 442.065 | -0.897 | 7.34E-06 | 0.000483 | hyper/hypo |
| 58 | HOXB3 | 64.558 | 1.547 | 0.001778 | 0.032915 | hyper/hypo |
| 59 | MAP3K14 | 651.224 | 0.774 | 0.001855 | 0.033836 | hyper/hypo |
| 60 | ABCA10 | 680.137 | 1.659 | 3.56E-07 | 3.76E-05 | hyper |
| 61 | RGMA | 713.185 | 1.09 | 0.003152 | 0.048447 | hyper |
| 62 | GPR68 | 82.398 | 2.522 | 7.14E-06 | 0.000474 | hyper |
| 63 | ASB2 | 7798.323 | 0.544 | 0.001501 | 0.029184 | hyper |
| 64 | SLC4A7 | 639.793 | -1.049 | 0.000475 | 0.012828 | hyper |
| 65 | XIRP1 | 137046.515 | -2.293 | 9.25E-11 | 2.78E-08 | hyper |
| 66 | RYBP | 744.833 | -1.001 | 3.75E-07 | 3.94E-05 | hyper |
| 67 | LRIG1 | 1669.42 | -0.598 | 0.001575 | 0.03009 | hyper |
| 68 | FAM107A | 1988.031 | 1.122 | 7.67E-05 | 0.003255 | hyper/hypo |
| 69 | PTH1R | 51.014 | 1.695 | 0.001873 | 0.033983 | hyper |
| 70 | MGLL | 2819.012 | 0.78 | 0.00014 | 0.00506 | hyper/hypo |
| 71 | JARID2 | 516.138 | -1.07 | 0.001551 | 0.02985 | hyper |
| 72 | CDKAL1 | 218.809 | -0.935 | 0.002364 | 0.039964 | hyper |
| 73 | HSP70.1 | 25138.632 | -1.779 | 0.000751 | 0.017753 | hyper |
| 74 | ITPR3 | 330.12 | 1.591 | 0.000636 | 0.016016 | hyper |
| 75 | BRD2 | 3780.596 | -0.647 | 0.000915 | 0.020422 | hyper |
| 76 | TAF4B | 196.769 | -1.306 | 0.002892 | 0.046007 | hyper |
| 77 | TTC39C | 91.069 | -1.653 | 0.001527 | 0.029547 | hyper |
| 78 | ANKRD12 | 1701.15 | -0.674 | 0.002806 | 0.04514 | hyper |
| 79 | FAM234A | 1040.525 | 0.724 | 2.66E-05 | 0.001371 | hyper |
| 80 | CTBP2 | 1359.975 | 0.738 | 3.31E-05 | 0.001648 | hyper/hypo |
| 81 | SFRP5 | 453.235 | 3.218 | 1.60E-12 | 6.84E-10 | hyper |
| 82 | PRKG1 | 3749.275 | -2.527 | 6.49E-20 | 8.85E-17 | hyper/hypo |
| 83 | IDO1 | 168.922 | -3.103 | 1.38E-05 | 0.000793 | hyper |
| 84 | SNX25 | 229.802 | -0.619 | 0.002691 | 0.043726 | hyper |
| 85 | MYOM2 | 14794.094 | -0.731 | 0.001951 | 0.034839 | hyper |
| 86 | ARID5B | 6662.111 | -2.417 | 1.81E-06 | 0.000155 | hyper |
| 87 | CHKA | 322.653 | -1.098 | 0.000694 | 0.016923 | hyper |
| 88 | MCEMP1 | 11.246 | -5.16 | 0.001091 | 0.023103 | hyper |
| 89 | B3GAT1 | 214.393 | 1.915 | 0.001999 | 0.035429 | hyper |
| 90 | SLC25A5 | 513.158 | -0.933 | 0.001762 | 0.032734 | hyper |
| 91 | TIPARP | 4032.243 | -1.378 | 4.86E-08 | 6.19E-06 | hypo |
| 92 | FAM124B | 36.74 | 1.814 | 0.001274 | 0.025715 | hypo |
| 93 | ARPC2 | 1773.764 | -0.505 | 0.002739 | 0.044363 | hypo |
| 94 | OSBPL6 | 520.614 | -1.229 | 3.05E-05 | 0.001551 | hypo |
| 95 | MSTN | 1518.609 | -2.034 | 3.97E-05 | 0.00191 | hypo |
| 96 | FOXO6 | 686.235 | 1.577 | 2.65E-06 | 0.000219 | hypo |
| 97 | PTPRF | 177.851 | 1.354 | 0.002079 | 0.036466 | hypo |
| 98 | PDE4B | 1508.734 | -2.196 | 5.80E-10 | 1.34E-07 | hypo |
| 99 | AGL | 9470.85 | -0.554 | 0.00113 | 0.023733 | hypo |
| 100 | SHE | 572.724 | 0.603 | 0.002892 | 0.046007 | hypo |
| 101 | PLXNA4 | 217.797 | 0.898 | 0.00196 | 0.034903 | hypo |
| 102 | FLNC | 169916.836 | -1.162 | 0.001547 | 0.029828 | hypo |
| 103 | DNAH11 | 177.455 | -1.159 | 0.000667 | 0.016394 | hypo |
| 104 | CLEC4D | 88.563 | -5.46 | 0.000345 | 0.00997 | hypo |
| 105 | SYNPO2 | 5104.388 | -0.747 | 0.000277 | 0.008463 | hypo |
| 106 | LDB2 | 202.691 | -1.138 | 0.000539 | 0.014134 | hypo |
| 107 | SGCD | 1738.15 | -0.504 | 0.002808 | 0.04514 | hypo |
| 108 | FNIP1 | 1467.572 | -1.497 | 1.05E-05 | 0.000635 | hypo |
| 109 | DOT1L | 3389.835 | -1.484 | 0.000289 | 0.008654 | hypo |
| 110 | ACP5 | 398.934 | 0.806 | 0.000485 | 0.013016 | hypo |
| 111 | PINX1 | 142.396 | -0.802 | 0.002025 | 0.035679 | hypo |
| 112 | EGR3 | 153.718 | -2.042 | 9.86E-11 | 2.90E-08 | hypo |
| 113 | PLN | 3124.856 | -0.885 | 6.86E-05 | 0.002969 | hypo |
| 114 | VGLL2 | 1325.558 | -1.196 | 5.92E-07 | 5.73E-05 | hypo |
| 115 | HS3ST5 | 34.939 | -1.772 | 0.000514 | 0.013679 | hypo |
| 116 | FOXO3 | 2898.879 | 0.752 | 0.000516 | 0.013712 | hypo |
| 117 | PLEKHG1 | 701.798 | -1.501 | 5.59E-05 | 0.002568 | hypo |
| 118 | AKAP12 | 12465.749 | -1.899 | 7.86E-05 | 0.003288 | hypo |
| 119 | DPF3 | 455.233 | 0.892 | 0.002296 | 0.039387 | hypo |
| 120 | SPTBN5 | 118.747 | 3.099 | 9.94E-05 | 0.003959 | hypo |
| 121 | TLE3 | 670.784 | -0.625 | 0.000375 | 0.010636 | hypo |
| 122 | CRIM1 | 1365.565 | 0.754 | 6.00E-05 | 0.002712 | hypo |
| 123 | SLC25A25 | 1858.14 | 2.58 | 3.74E-24 | 1.12E-20 | hypo |
| 124 | PKIG | 636.806 | -1.464 | 0.000115 | 0.004323 | hypo |
| 125 | YWHAZ | 2306.312 | -0.651 | 0.002567 | 0.042169 | hypo |
| 126 | SH2B3 | 1088.647 | -0.938 | 0.000218 | 0.007067 | hypo |
| 127 | GALNT9 | 9.708 | 4.709 | 0.00254 | 0.041944 | hypo |
| 128 | BANP | 289.196 | 1.012 | 1.46E-05 | 0.000829 | hypo |
| 129 | CBFA2T3 | 1588.055 | 1.113 | 2.81E-05 | 0.001443 | hypo |
| 130 | CDC42EP5 | 208.874 | 0.958 | 5.80E-06 | 0.000414 | hypo |
| 131 | YPEL2 | 902.073 | 0.887 | 0.000861 | 0.01947 | hypo |
| 132 | SLC16A5 | 706.854 | 1.306 | 5.64E-05 | 0.002584 | hypo |
| 133 | CPEB4 | 1308.273 | -1.019 | 1.20E-07 | 1.42E-05 | hypo |
| 134 | PRICKLE2 | 129.965 | 1.462 | 2.29E-05 | 0.001192 | hypo |
| 135 | F13A1 | 799.609 | -2.159 | 5.40E-05 | 0.002496 | hypo |
| 136 | SETBP1 | 506.85 | -1.113 | 5.42E-05 | 0.002496 | hypo |
| 137 | MAPK8IP3 | 177.191 | 0.74 | 0.002967 | 0.046606 | hypo |
| 138 | TRAF7 | 744.438 | 0.69 | 0.000298 | 0.008898 | hypo |
| 139 | EMP2 | 3937.98 | 1.157 | 0.000105 | 0.004151 | hypo |
| 140 | ARHGEF10 | 549.818 | 0.791 | 0.000777 | 0.018082 | hypo |
| 141 | PANX1 | 254.254 | -0.923 | 0.000774 | 0.01807 | hypo |
| 142 | RASGRP2 | 148.843 | 1.259 | 0.001317 | 0.026363 | hypo |
| 143 | PRRG3 | 249.478 | 1.736 | 0.00065 | 0.016105 | hypo |
| 144 | TSC22D3 | 13983.422 | 0.816 | 0.00109 | 0.023103 | hypo |
| 145 | ABL1 | 1695.442 | -0.882 | 0.00017 | 0.00601 | hypo |
| 146 | ETS1 | 2813.180 | -1.277 | 4.0091E-08 | 5.22299E-06 | hyper |
| 147 | IL2RA | 46.579 | -1.594 | 0.00125 | 0.02537 | hyper/hypo |
| 148 | ITGA8 | 147.803 | 1.251 | 0.00046 | 0.01255 | hypo |
| 149 | NR4A1 | 3230.685 | -1.677 | 6.67698E-06 | 0.00045 | hypo |
| 150 | RELB | 243.139 | -1.530 | 3.32355E-05 | 0.00164 | hypo |
| 151 | TET2 | 2363.927 | -1.477 | 4.41725E-05 | 0.00211 | hypo |
| 152 | LOC102170232 | 527.260 | -0.802 | 0.00094 | 0.02075 | hypo |
| 153 | LOC102171650 | 158.947 | 1.403 | 3.21774E-08 | 4.38256E-06 | hypo |
| 154 | LOC102173333 | 148.248 | 1.223 | 0.00066 | 0.01635 | hypo |
| 155 | LOC102177850 | 218.745 | -2.796 | 7.37635E-06 | 0.00048 | hyper |
| 156 | LOC102178109 | 709.665 | 1.069 | 0.00088 | 0.01979 | hypo |
| 157 | LOC102182683 | 102.227 | -5.548 | 4.92787E-11 | 1.50672E-08 | hypo |
| 158 | LOC102185525 | 26.566 | -6.435 | 3.38947E-06 | 0.00026 | hyper |
| 159 | LOC102186926 | 40.295 | 3.137 | 0.00170 | 0.03184 | hyper |
| 160 | LOC102188072 | 8581.983 | -2.208 | 4.45603E-07 | 4.42121E-05 | hyper |
| 161 | LOC106503997 | 13.887 | 3.055 | 0.00090 | 0.02023 | hyper |
| 162 | LOC108633505 | 1994.764 | 1.441 | 0.00012 | 0.00460 | hypo |
| 163 | LOC108635088 | 9.054 | 6.500 | 1.03119E-05 | 0.00062 | hyper |
| 164 | LOC108635288 | 226.065 | -1.108 | 8.90519E-05 | 0.00362 | hyper |
| 165 | LOC108635449 | 280.589 | -1.823 | 3.54024E-05 | 0.00173 | hypo |
| 166 | LOC108635459 | 34.002 | -3.065 | 0.00104 | 0.02234 | hyper |

Symbol meant the gene name; BaseMean was the average expression of gene; padj meant adjusted *P* value; methystat meant hyper and hypo DMR.

**Table S7** The PPI node score of 11 hub genes

| ID | Symbol | node_degree |
| --- | --- | --- |
| 1 | ADCY5 | 6 |
| 2 | AKT2 | 7 |
| 3 | EZH2 | 10 |
| 4 | FOXO3 | 8 |
| 5 | FOXO6 | 4 |
| 6 | GNAO1 | 4 |
| 7 | IRS2 | 4 |
| 8 | PRKG1 | 7 |
| 9 | RUNX1 | 9 |
| 10 | ZBTB16 | 4 |
| 11 | NR4A1 | 5 |

The hub genes were selected based on their location closer to center and their node score more or equal to 4, and the connection between genes have becoming stronger when the score was getting higher.

**Table S8** The methylation sites of 11 hub genes

| Gene Name | chr | Start-end | length | n  CG | hybrid_m_  meanMethy | hainan_m_  meanMethy | diff.  Methy | area  Stat | C_  context | region |
| --- | --- | --- | --- | --- | --- | --- | --- | --- | --- | --- |
| ADCY5 | 1 | 67571853-  67571968 | 116 | 50 | 0.040 | 0.007 | 0.033 | 269.832 | CHH | intron |
| ADCY5 | 1 | 67575236-  67575367 | 132 | 46 | 0.027 | 0.004 | 0.022 | 211.826 | CHH | intron |
| AKT2 | 18 | 50570819-  50570932 | 114 | 36 | 0.198 | 0.073 | 0.125 | 169.474 | CG | intron |
| AKT2 | 18 | 50543407-  50543548 | 142 | 17 | 0.271 | 0.446 | -0.175 | -76.477 | CG | intron |
| AKT2 | 18 | 50543685-50543793 | 109 | 17 | 0.389 | 0.578 | -0.188 | -85.289 | CG | intron |
| EZH2 | 4 | 8656507-  8656572 | 66 | 13 | 0.857 | 0.742 | 0.115 | 57.678 | CG | exon |
| EZH2 | 4 | 8656507-  8656572 | 66 | 13 | 0.857 | 0.742 | 0.115 | 57.678 | CG | intron |
| FOXO3 | 9 | 28854796-  28854906 | 111 | 11 | 0.337 | 0.570 | -0.233 | -51.493 | CG | intron |
| FOXO6 | 3 | 16003042-  16003289 | 248 | 69 | 0.213 | 0.384 | -0.171 | -421.844 | CG | exon |
| GNAO1 | 18 | 25746296-25746384 | 89 | 11 | 0.438 | 0.262 | 0.176 | 46.908 | CG | intron |
| IRS2 | 12 | 1639946-  1640035 | 90 | 23 | 0.953 | 0.883 | 0.070 | 96.497 | CG | exon |
| PRKG1 | 26 | 43948478-  43948576 | 99 | 10 | 0.417 | 0.097 | 0.319 | 57.372 | CG | intron |
| PRKG1 | 26 | 43786820-  43786872 | 53 | 6 | 0.001 | 0.029 | -0.028 | -28.496 | CHG | intron |
| RUNX1 | 1 | 146898385-  146898463 | 79 | 7 | 0.844 | 0.609 | 0.235 | 30.236 | CG | intron |
| ZBTB16 | 15 | 57615791-  57615854 | 64 | 6 | 0.252 | 0.079 | 0.172 | 27.673 | CG | intron |
| ZBTB16 | 15 | 57616855-  57616923 | 69 | 30 | 0.026 | 0.004 | 0.022 | 134.618 | CHH | intron |
| ZBTB16 | 15 | 57745058-  57745157 | 100 | 10 | 0.207 | 0.446 | -0.239 | -51.069 | CG | intron |
| NR4A1 | 5 | 27535256-  27535416 | 161 | 18 | 0.184 | 0.390 | -0.207 | -93.880 | CG | intron |
| NR4A1 | 5 | 27535256-27535416 | 161 | 18 | 0.184 | 0.390 | -0.207 | -93.880 | CG | promoter |

**Table S9** The expressions and methylation levels of 11 genes with correlations in hypo DMRs

| Gene name | log_2_FoldChange | diff.Methy |
| --- | --- | --- |
| *AKT2* | 0.609 | -0.175 |
| *FOXO3* | 0.752 | -0.233 |
| *FOXO6* | 1.577 | -0.171 |
| *PRKG1* | -2.527 | -0.028 |
| *ZBTB16* | 0.963 | -0.239 |
| *NR4A1* | -1.678 | -0.207 |
| *ITGA8* | 1.251 | -0.570 |
| *MAP3K14* | 0.774 | -0.215 |
| *RELB* | -1.531 | -0.027 |
| *IL2RA* | -1.595 | -0.149 |
| *FLNC* | -1.162 | -0.031 |

**Table S10** The growth traits of hybrid goats and Hainan black goats in LEA

| Sample ID | Species | Weight (kg) | LEA height (cm) | LEA width (cm) | LEA area (cm^2^) |
| --- | --- | --- | --- | --- | --- |
| hybrid1 | Hybrid | 25.5 | 4.91 | 3.19 | 10.96 |
| hybrid2 | Hybrid | 25.0 | 5.00 | 2.90 | 10.15 |
| hybrid3 | Hybrid | 22.6 | 5.35 | 2.88 | 10.79 |
| Hainan1 | Hainan | 16.7 | 4.03 | 3.13 | 8.83 |
| Hainan2 | Hainan | 17.0 | 4.37 | 2.92 | 8.93 |
| Hainan3 | Hainan | 18.6 | 4.67 | 2.49 | 8.14 |

The sample ID hybrid meant cross-fertilized offspring of Nubia goat and Hainan black goat. Hainan meant Hainan black goat.

LEA area= LEA height × LEA width × 0.7

**Table S11** The primer sequences for RT-qPCR

| ID | Genes | Primer sequences |
| --- | --- | --- |
| 1 | ADCY5-F | CGGTGTTGTATTCGAGTCC |
|  | ADCY5-R | CAGGTTGGGAGCAAGATG |
| 2 | AKT2-F | CCGCTTCACAAACTCTTC |
|  | AKT2-R | AACTGGAGACCAAGTGTT |
| 3 | FOXO3-F | TAGAACTCAGTGTGGTGC |
|  | FOXO3-R | CGCTTTGAAAGGAAGGATG |
| 4 | FOXO6-F | CTCCGAATCCTCAGTATCAT |
|  | FOXO6-R | CAGTGGGAAAGTGTCAATAG |
| 5 | PRKG1-F | TGCTTCCTCTGAGTTCTT |
|  | PRKG1-R | GCAAGGGCAGATAAAGAG |

F: forward primer; R: reverse primer.

**Supplementary figures**


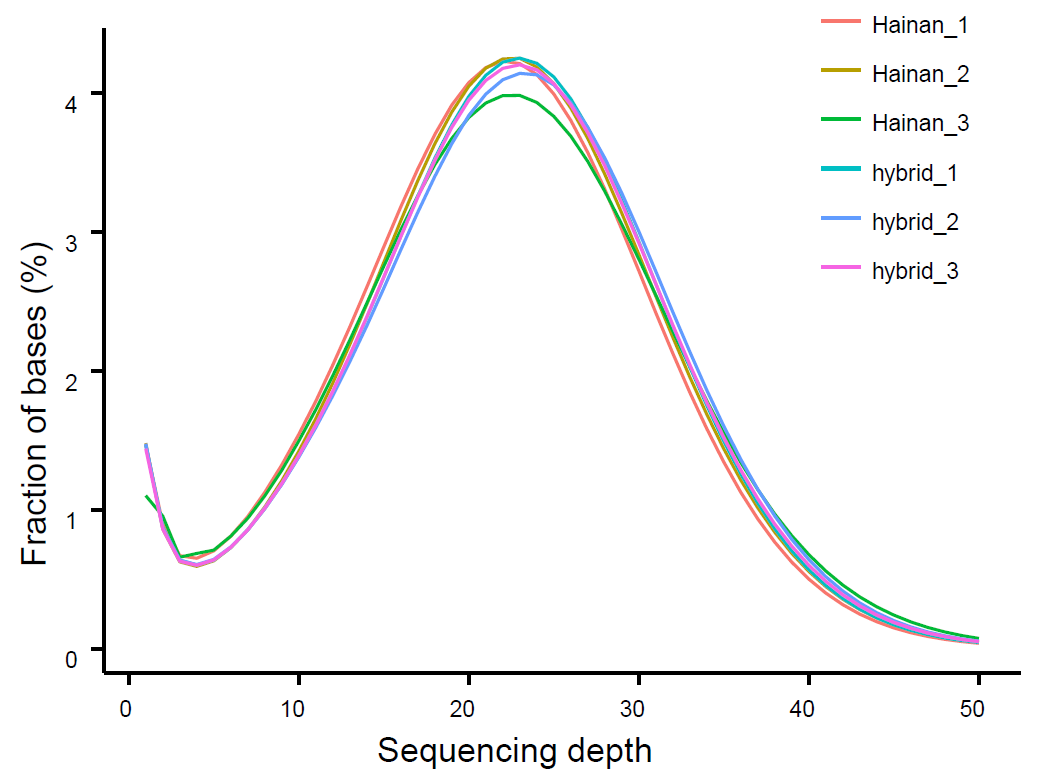


**Fig. S1** The sequencing depth distribution of all bases for Hainan black goats and hybrid goats


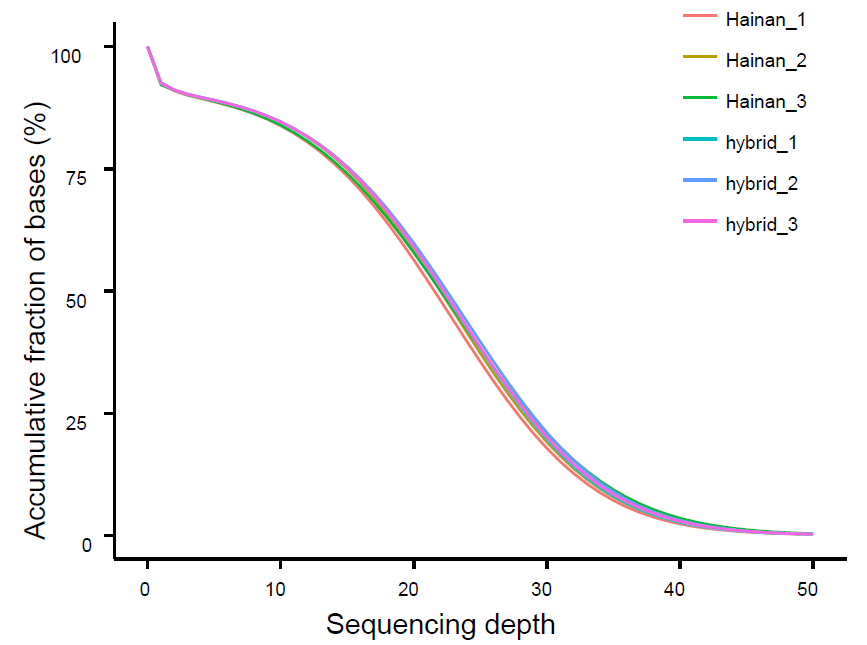


**Fig. S2** The sequencing depth distribution of base accumulative in Hainan black goats and hybrid goats. The x-axis meant the sequencing depth; the y-axis meant the percentage of accumulative fraction of bases.

**Fig. S3** The average CG context number of chromosomes in Hainan black goats and hybrid goats. Blue color meant Hainan black goats, and orange color meant Hybrid goats.


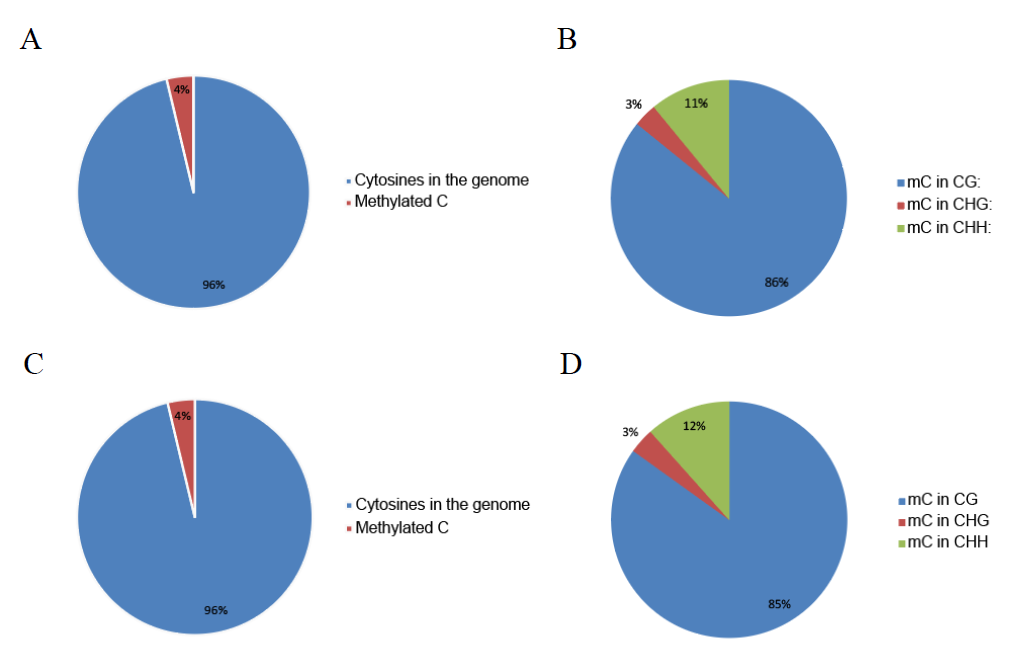


**Fig. S4** The classification of mean mC proportions in LDM. **(A)** The mean mC proportion of Hainan black goats. **(B)** The mean proportion of three mC context (CG, CHG, CHH) of Hainan black goats. **(C)** The mean mC proportion of hybrid goats. **(D)** The mean proportion of three mC context (CG, CHG, CHH) of hybrid goat.

**
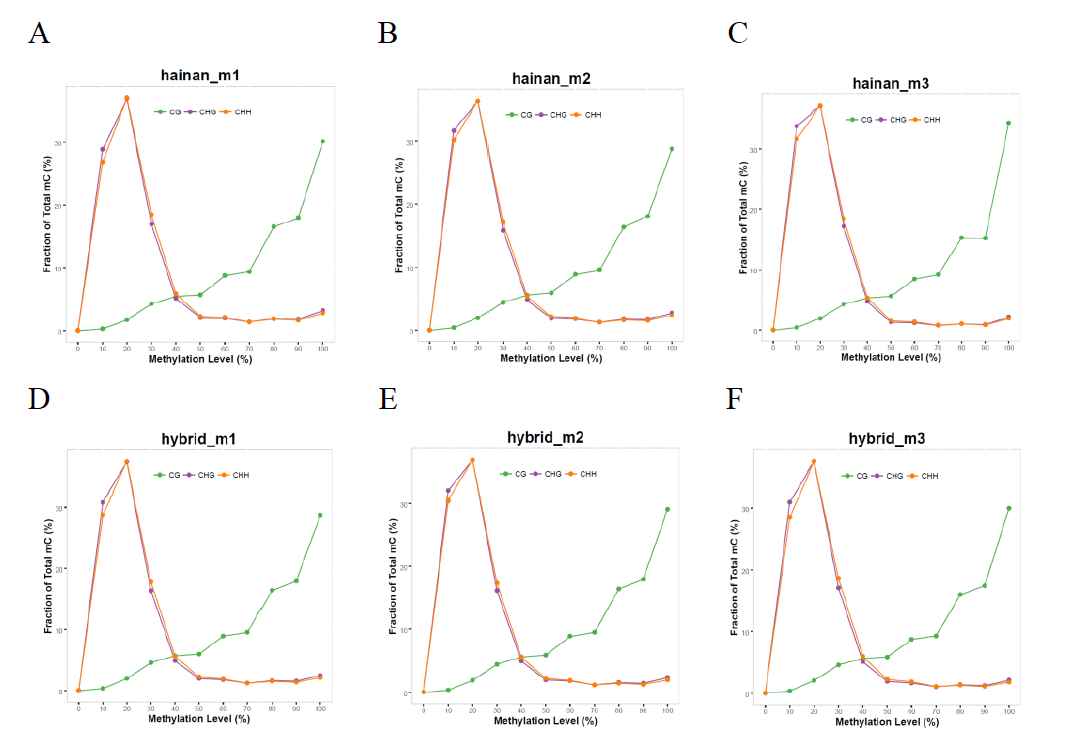
Fig. S5** The methylation level distribution of mC contexts. Three mC contexts showed in Hainan black goats **(A) (B) (C)** and hybrid goats **(D) (E) (F)**. The x-axis meant the methylation level; the y-axis meant the proportion of the three mC contexts (CG, CHG, and CHH) on different methylation level region.


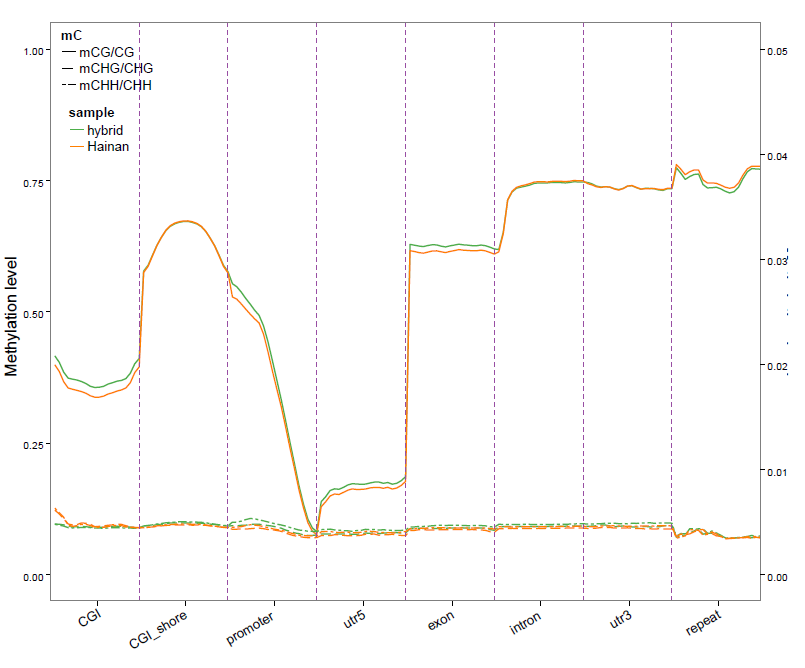


**Fig. S6** The average mC levels in different functional regions. The figure showed the average mC levels in functional regions (promoter, exon, intron, CGI, CGI shore, UTR3, UTR5, repeat) of LDM for Hainan black goats and hybrid goats.


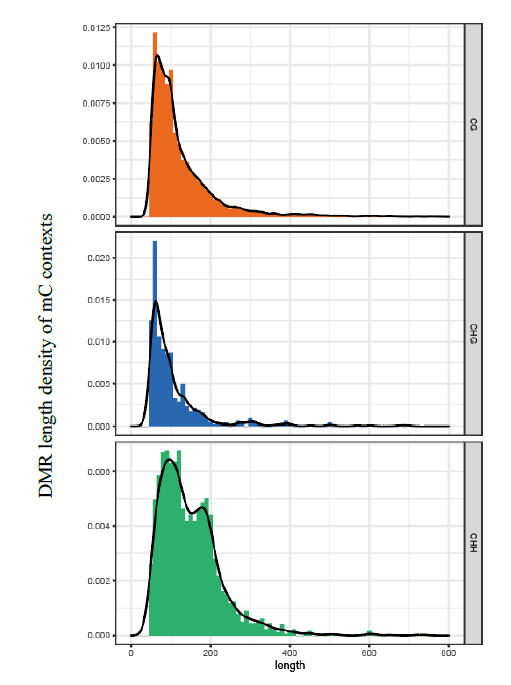


**Fig. S7** The length density distribution of three mC contexts in LDM.


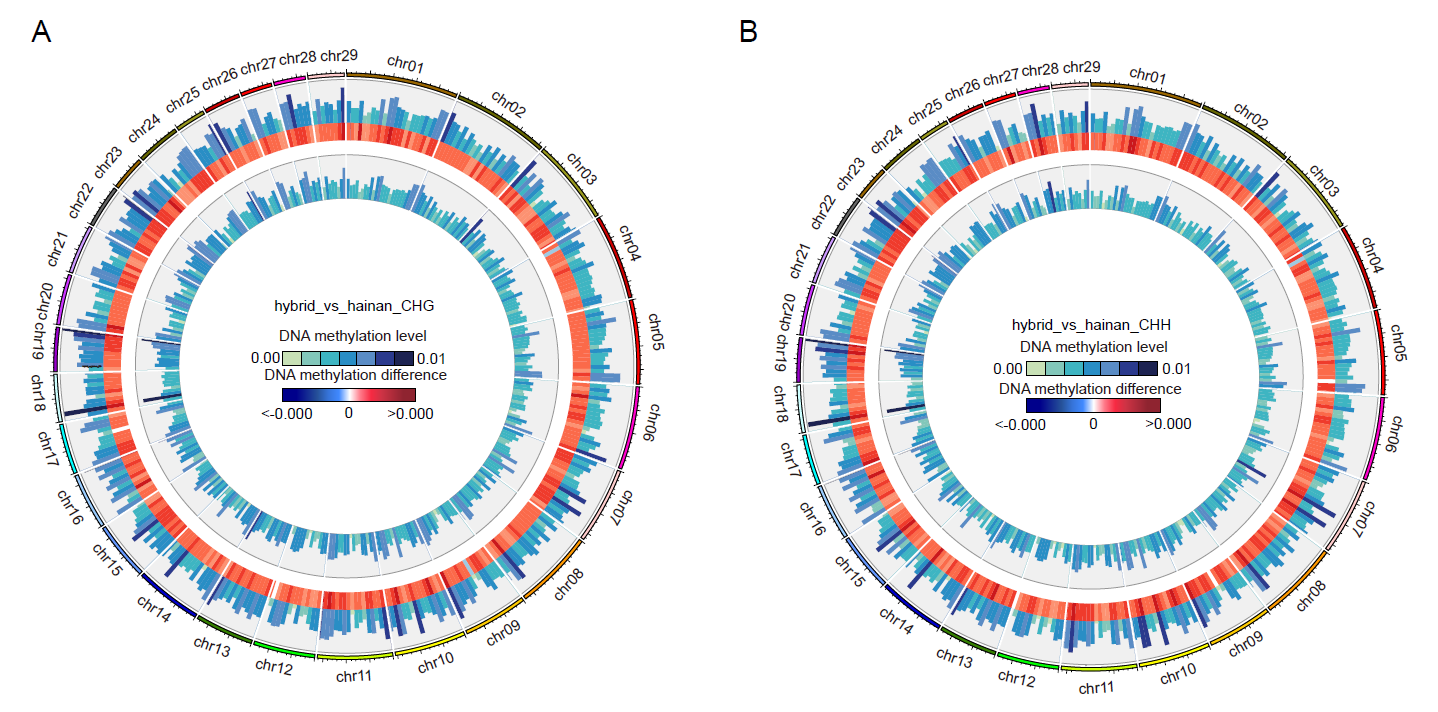


**Fig. S8** The DMR analysis of LDM between Hainan black goats and hybrid goats. The circle outermost layer and the innermost layer were the DNA methylation level of hybrid goat CHG **(A)** and CHH **(B)** content, separately. The middle layer was the differences of the DNA methylation level between two goat species. The circos figure bin was 6553600 bp, and the dark blue of DNA methylation difference meant the significantly lower DNA methylation level of hybrid goats compared with that of Hainan black goats, while the red color meant the significantly higher DNA methylation level.


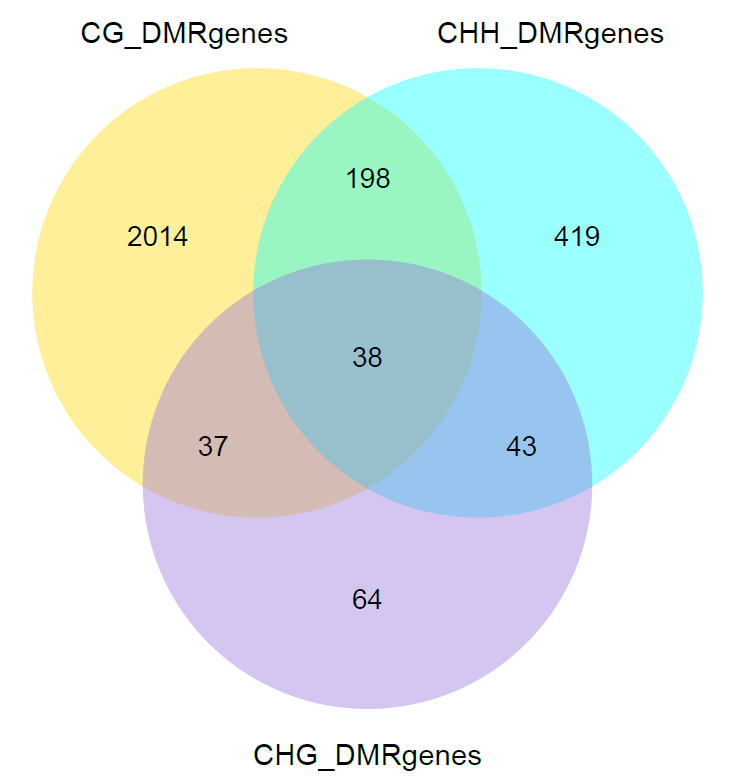


**Fig. S9** The number of annotated genes in DMRs. The yellow circle meant the gene number of CG; the purple circle meant the gene number of CHG; the blue circle meant the gene number of CHH.


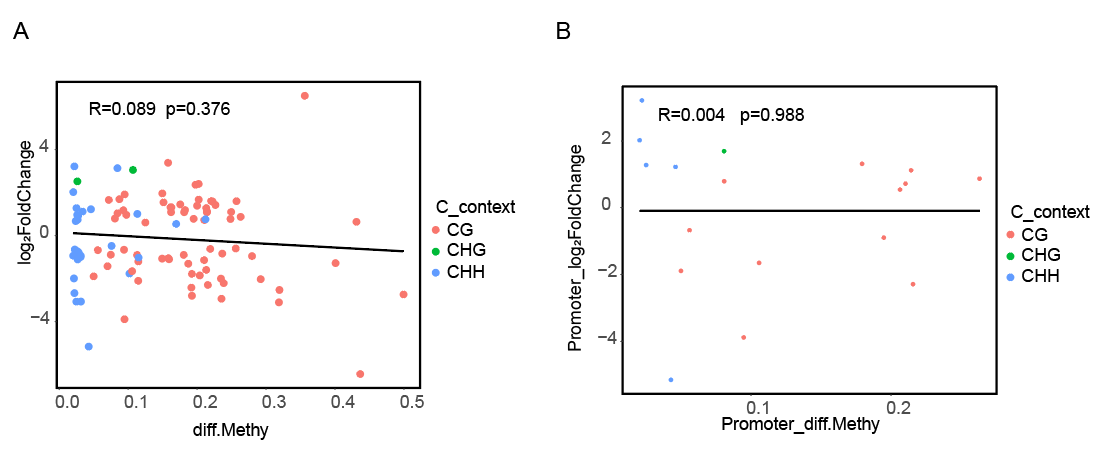


**Fig. S10** The correlations between methylation levels and the expression of DEGs in hyper DMR. **(A)** The correlations between the expression and methylation level of 100 genes from the intersection of hyper DMR and DEGs; **(B)** the correlations between the expression and methylation level of the genes with promoters.


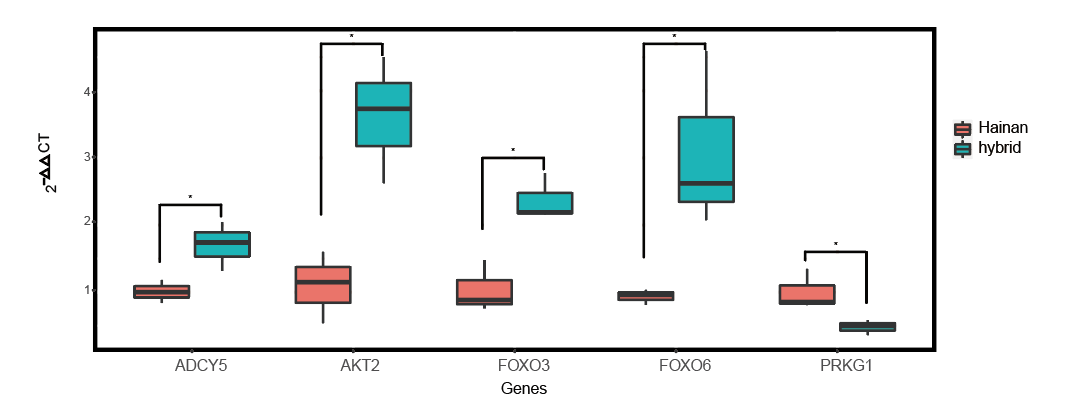


**Fig. S11** The RT-qPCR results of 5 most important genes. The signal * meant *P* < 0.05
